# Gaps in the welfare state: A role-based model of poverty risk in the U.S.

**DOI:** 10.1371/journal.pone.0284251

**Published:** 2023-04-13

**Authors:** Seth A. Berkowitz, Deepak Palakshappa

**Affiliations:** 1 Division of General Medicine and Clinical Epidemiology, Department of Medicine, University of North Carolina at Chapel Hill School of Medicine, Chapel Hill, North Carolina, United States of America; 2 Cecil G. Sheps Center for Health Services Research, University of North Carolina at Chapel Hill, Chapel Hill, North Carolina, United States of America; 3 Section of General Internal Medicine, Department of Internal Medicine, Wake Forest University School of Medicine, Winston-Salem, North Carolina, United States of America; 4 Section of General Pediatrics, Department of Pediatrics, Wake Forest University School of Medicine, Winston-Salem, North Carolina, United States of America; 5 Department of Epidemiology and Prevention, Division of Public Health Sciences, Wake Forest University School of Medicine, Winston-Salem, North Carolina, United States of America; Universite Paris Pantheon-Assas, FRANCE

## Abstract

**Background:**

Research clearly demonstrates that income matters greatly to health. However, income distribution and its relationship to poverty risk is often misunderstood.

**Methods:**

We provide a structural account of income distribution and poverty risk in the U.S., rooted in the ‘roles’ that individuals inhabit with relation to the ‘factor payment system’ (market distribution of income to individuals through wages and asset ownership). Principal roles are child, older adult, and, among working-age adults, disabled individual, student, unemployed individual, caregiver, or paid laborer. Moreover, the roles of other members of an individual’s household also influence an individual’s income level. This account implies that 1) roles other than paid laborer will be associated with greater poverty risk, 2) household composition will be associated with poverty risk, and 3) income support policies for those not able to engage in paid labor are critical for avoiding poverty. We test hypotheses implied by this account using 2019 and 2022 U.S. Census Current Population Survey data. The exposure variables in our analyses relate to roles and household composition. The outcomes relate to income and poverty risk.

**Results:**

In 2019, 40.1 million individuals (12.7% of the population) experienced poverty under the U.S. Census’ Supplemental Poverty Measure. All roles other than paid laborer were associated with greater poverty risk (p < .001 for all comparisons). Household composition, particularly more children and disabled working-age adults, and fewer paid laborers, was also associated with greater poverty risk (p < .001 for all comparisons). Five key policy areas—child benefits, older-age pensions, disability and sickness insurance, unemployment insurance, and out-of-pocket healthcare spending—represented gaps in the welfare state strongly associated with poverty risk.

**Conclusions:**

The role one inhabits and household composition are associated with poverty risk. This understanding of income distribution and poverty risk may be useful for social policy.

## Introduction

Nearly 100 years ago, Isaac Rubinow, a physician and early advocate of social insurance in the U.S., wrote: “in the enormous literature dealing with the phenomena of social life, largely descriptive, historical, or speculative, there are surprisingly few “scientific laws”, or rules of causal relationship. But among the few there are none which could claim a greater scientific accuracy than the following two: Poverty causes ill health; ill health causes poverty” [1]. Research since that time has only further linked poor health with poverty specifically and issues of income distribution more broadly [2–7]. Indeed, in a seminal paper, Link and Phelan identified income as a ‘fundamental cause of health’ [8]. Poverty, which can be thought of as capability deprivation resulting from a location at the extreme lower end of a society’s income distribution, is a specific manifestation of the larger role that distributive institutions play in society [9–11].

Because the social epidemiological and health equity research that connects poor health and income distribution is so well established, further research demonstrating this connection is not needed. Instead, for progress in this area, it would be helpful to focus on how income is distributed or poverty occurs, which epidemiological research often does not do. For example, prominent reports, like the Agency for Healthcare Research and Quality’s (AHRQ) National Healthcare Quality and Disparities Report, Healthy People 2030, Centers for Medicare and Medicaid’s (CMS) Framework for Health Equity, and the National Academy of Medicine’s Perspectives on Health Equity and Social Determinants of Health, all note the association between low income and poor health, but none provide an account of income distribution [12–15]. As such, work in this field often provides little detail as to how income is distributed as it is, or why some individuals are at much higher risk of experiencing poverty than others.

In the absence of a clear account of how poverty predictably emerges from a society’s distributive institutions, people may turn to accounts that emphasize the behavioral deficiencies or cultural inferiority of those who experience poverty [10, 16, 17]. Given the history of racial injustice in the U.S., these accounts are often racialized [18, 19].

Studies in the field of economics have examined how poverty can be conceived of as capability deprivation, and result from resource allocation within a society [9, 20]. This has led to work examining how income support and other policies can best address poverty risk [21, 22].

Drawing on this past work, we present a structural analysis of income distribution and poverty risk [23–25]. The analysis starts with two stylized facts. First, the principal institution of income distribution in a market-based economy, such as the U.S., is the ‘factor payment system’. In the factor payment system, those who perform paid labor and those who own productive assets receive ‘factor income’ (so called because in economics labor, land, and capital are the ‘factors’ of production). Factor income can be contrasted with ‘transfer income’, income distributed without exchange of goods or services (as through government income support policies). Total income consists of factor income plus transfer income, minus tax liabilities.

The second stylized fact is that individuals commonly pool resources as households (of course, single-person households do occur), and households often serve as the unit of economic analysis.

Since the factor payment system is the primary mechanism of income distribution, one’s relation to the factor payment system, and in particular to the labor market, is likely to be closely connected with the amount of income received, and one’s poverty risk.

An individual’s relation to the labor market can be thought of as a ‘role’ that they inhabit [26]. Characterizing these roles begins with age. Below and above a certain age, one typically cannot and/or should not work to earn factor income. The specific ages chosen are historically contingent and result from political contestation, but it is widely recognized that children and older adults do not have a responsibility to perform paid labor. In contrast, there is often a default expectation of performing paid labor for ‘working-age’ adults. Nevertheless, there are certain exceptions—disability, pursuing education full time, unemployment, and caregiving (i.e., performing unpaid but socially necessary labor in caring for children or adults unable to care for themselves) are typically seen as valid reasons why one cannot engage in paid labor. Taken together, these roles—child, older adult, disabled working-age adult, student, unemployed working-age adult, caregiver, paid laborer, and working-age adults who do not fill one of the other roles, help define one’s relationship to the factor payment system.

The term ‘role’ is meant to emphasize that these are not distinct classes of people—most individuals will fill more than one role over the course of their life, and people in each role may have little else in common with each other. Roles are states people are in within a given social structure, not traits that they have. Similarly, poverty is a material state of capability deprivation, not an identity.

Although an individual’s role is of principal importance to income distribution and poverty risk, the second stylized fact—that individuals often aggregate into households—means that an individual’s personal role does not tell the whole story: the roles of others in an individual’s household matter as well.

This account of income distribution is structural, as opposed to behavioral or cultural, in that it emphasizes the distributive institutions within a society—those rules and practices that distribute income—as creating ‘spaces’ within a social structure for people to become poor [23, 27]. It deemphasizes explanations that focus on individual behavior or the cultural characteristics of categories of individuals. Further, it has testable implications. First, it suggests that those with roles other than paid laborer will have a higher prevalence of poverty. Second, it suggests that even among those who are engaging in paid labor, their poverty risk will be greater if they have more members of their household who are not—for example, children or disabled working-age adults. Third, it suggests that, because all individuals need the power to consume goods and services allocated by market mechanisms, but not all individuals receive factor payments, income support policies that transfer purchasing power to those who do not receive factor payments are of vital importance [28].

The aspects of the state responsible for income support policies are sometimes known as the ‘welfare state’ [22, 29]. In this manuscript, we use comprehensive data from the U.S. Census Bureau’s Current Population Survey (CPS) Annual Social and Economic Supplement (ASEC [sic]) to examine empirically the proposed model of income distribution and poverty. We test hypotheses relating poverty risk to the roles we have characterized, and to household composition. The exposure variables in our analyses relate to roles and household composition. The outcomes relate to income and poverty risk. Further, we quantify specific gaps in the welfare state in the U.S., focusing on five key areas that prior research in the field of economics has indicated may be important to consider: child benefits, older age pensions, the short and long-term disability and sickness insurance system (including paid leave from work), the unemployment insurance system, and out-of-pocket healthcare spending [30–40]. The overarching goal of this paper is to make sense of data on poverty and income distribution in a way that can inform social policy.

## Materials and methods

### Data sources

Data for this study came from the 2019 and 2022 CPS ASEC, conducted by the U.S. Census Bureau [41]. ASEC data reflects the economic conditions of the prior calendar year—e.g., 2019 ASEC data reflects 2018. The 2019 ASEC was the primary dataset used for this study as it was the most recent year before the disruptions of the COVID-19 pandemic. 2022 ASEC data were used to check the robustness of the primary study findings, but were not the primary data used because many pandemic-era income support policies, such as stimulus payments and the expanded Child Tax Credit, were still in effect in 2021 (the year covered by the 2022 ASEC data), but have since expired. This means that 2019 ASEC data reflect a policy environment more similar to the current policy environment than 2022 ASEC data. 2020 and 2021 ASEC data were not used owing to the disruptions in data collection caused by the COVID-19 pandemic in both years, and pandemic income support policies reflected in the 2021 ASEC data.

All individuals within the ASEC were included in this study, without exclusions. The institutional review board at the University of North Carolina at Chapel Hill determined that this secondary analysis of deidentified data did not constitute human subjects research, and thus waived the requirement for informed consent.

### Income and poverty

There are many possible income concepts that can be used for research related to income and health. In general, the concept used should include factor income, transfer income, and tax liabilities [42–44]. Further, the income concept used should include both money and non-money (e.g., benefits from the Supplemental Nutrition Assistance Program [SNAP]) sources of income. In this study, we used the ‘supplemental poverty measure resources’ income concept, as defined and estimated by the U.S. Census Bureau, which has these features [41, 45]. Moreover, owing to pooling of resources, income should include all household sources, and should be ‘equivalized’ for household size to permit comparisons between individuals who live in households of varying size. The method of equivalization used in this study is to divide the household income by the square root of the number of household members [42].

Assessment of poverty status involves comparing the income concept used to a threshold. One common threshold in the U.S. is the official poverty measure (OPM). However, the OPM does not count many forms of non-money income used for poverty-fighting (e.g., SNAP), and thus has serious drawbacks in understanding the relationship between income and poverty [46]. Therefore, we instead used the U.S. Census Bureau’s Supplemental Poverty Measure (SPM) threshold, using the variable within ASEC that categorizes a household (‘SPM resource unit’) as poor if their SPM resources are at or below the relevant threshold for their area of residence and household size, and not poor if they are above it. We refer to this as experiencing poverty under an absolute threshold. Internationally, ‘relative’ poverty measures are more commonly used. These relate a household’s income to, most typically, 50% of a country’s median household income. Such an approach is used, for example, in comparisons of poverty across countries presented by OECD [47]. To create a relative measure for this study, we calculated the equalivalized SPM resources that each individual within a household had, and compared that to 50% of the national median equalivalized SPM resources for a given study year. Values at or below 50% of the median were categorized as experiencing poverty under a relative threshold, and values above 50% were categorized as not experiencing poverty under a relative threshold.

### Roles

Given that earned income from paid labor is the primary method of factor income distribution in the U.S., we used variables within the ASEC to categorize individuals into ‘roles’ that relate to the reasons they would or would not be expected to engage in paid labor [26, 48]. First, we divided individuals into three categories based on age: children (under 18 years of age), working-age adults (age 18 to 64 years), and older adults (65 years of age or greater). Next, we sub-categorized working-age adults with regard to paid labor status using self-reported reasons. Working-age adults who engaged in paid labor at any time in the survey year were categorized as ‘paid laborers’. Working-age adults who reported that they did not engage in paid labor at all during the survey year were categorized as ‘disabled’ if they gave as the main reason for not engaging in paid labor “ill or disabled”, ‘student’ if they gave as the main reason “going to school”, ‘caregiver’ if they gave as the main reason “taking care of home”, ‘unemployed’ if they gave as the main reason “could not find work”, and ‘other’ if they did not give any of those reasons. All ASEC participants were categorized into one of these eight mutually exclusive roles.

### Other key variables

Other key variables extracted from ASEC data included demographic characteristics (including race and ethnicity as categorized within ASEC, which was used to indicate the potential experience of racism), experiences with the labor market such as job loss due to sickness or unemployment, and variables related to household composition. An important category of variables related to income source. These variables included factor income from labor (such as wages and salaries), factor income from capital ownership (such as interest and dividend income), income from retirement sources, disability income, unemployment income, income for education, child support, and income from means-tested programs. Construction of these variables is discussed in the **Technical Appendix in** S1 File.

### Statistical analyses

Statistical testing for comparisons between groups were made using t-tests and chi-squared tests, and predictive margins following fitting logistic regression models, as appropriate to the data type (e.g., continuous or categorical variables). All analyses utilized the representativeness weights provided by the U.S. Census Bureau, and variances were estimated, as recommended by the U.S. Census, using balanced repeated replicate estimation, with the replicates provided as part of ASEC [41]. Analyses were conducted in SAS version 9.4, Stata 16.1, and R version 4.2.0. A p-value < 0.05 was taken to indicate statistical significance.

## Results

In the 2019 ASEC, there were 180,101 participants (Table 1), representing 317 million individuals. By roles, there were 71.5 million (22.6% of the population) children under age 18, 53.0 million (16.7% of the population) adults aged 65 and older, and 192.2 million (60.7% of the population) working-age adults (age 18–64). Of working-age adults, 11.7 million individuals (3.7% of the entire population) reported being disabled, 8.7 million individuals (2.7%) reported being full-time students, 12.8 million individuals (4.0%) reported being a caregiver, 1.5 million individuals (0.5%) reported long-term unemployment, and 9.7 million individuals (3.1%) reported other reasons. There were 147.9 million working-age adults (46.7% of the entire population) in the paid labor force, meaning that over half of individuals in the U.S. are not expected to receive factor income from labor.

**Table 1:** Demographics, 2019 ASEC data.

	Overall	Poverty Under Absolute Threshold			Poverty Under Relative Threshold		
		Not in Poverty	In Poverty	p-value	Not in Poverty	In Poverty	p-value
Weighted N	316,766,849	276,662,646	40,104,203		258,574,533	58,192,316	
	N (%) or Mean (SD)	N (%) or Mean (SD)	N (%) or Mean (SD)		N (%) or Mean (SD)	N (%) or Mean (SD)	
Age, y	39.25 (23.05)	39.37 (22.90)	38.42 (24.02)	<.001	39.04 (22.59)	40.16 (24.96)	<.001
Female	51.41 (162863548)	51.02 (141143001)	54.16 (21720547)	<.001	50.70 (131104121)	54.58 (31759426)	<.001
Race and Ethnicity				<.001			<.001
Non-Hispanic White	60.92 (192980715)	63.70 (176231761)	41.76 (16748955)		63.86 (165137373)	47.85 (27843342)	
Non-Hispanic Black	11.76 (37247304)	10.72 (29669169)	18.90 (7578135)		10.29 (26619838)	18.26 (10627466)	
Non-Hispanic American Indian and Alaskan Native	0.80 (2543850)	0.75 (2075314)	1.17 (468536)		0.67 (1725503)	1.41 (818347)	
Non-Hispanic Asian	6.24 (19751160)	6.15 (17005231)	6.85 (2745929)		6.47 (16731371)	5.19 (3019789)	
Non-Hispanic Native Hawaiian and Other Pacific Islander	0.30 (935242)	0.28 (779334)	0.39 (155908)		0.29 (753536)	0.31 (181706)	
Non-Hispanic Multi-Racial	1.93 (6099844)	1.92 (5323977)	1.93 (775867)		1.93 (4980382)	1.92 (1119462)	
Hispanic, any race	18.06 (57208733)	16.47 (45577860)	29.00 (11630874)		16.49 (42626529)	25.06 (14582204)	
Education				<.001			<.001
Child, education incomplete	18.65 (59079350)	18.47 (51095030)	19.91 (7984321)		18.34 (47429189)	20.02 (11650161)	
Less than HS Diploma	11.90 (37699465)	10.49 (29014442)	21.66 (8685023)		9.96 (25742470)	20.55 (11956995)	
HS Diploma or GED	21.88 (69312515)	21.22 (58717336)	26.42 (10595179)		20.59 (53251435)	27.60 (16061080)	
Greater than HS Diploma	47.57 (150675519)	49.82 (137835838)	32.02 (12839681)		51.11 (132151439)	31.83 (18524080)	
Marital Status				<.001			<.001
Married—Civilian Spouse Present	40.49 (128258668)	42.87 (118595982)	24.09 (9662684)		43.98 (113723421)	24.98 (14535246)	
Married—Armed Forces Spouse Present	0.24 (761104)	0.26 (725787)	0.09 (35317)		0.27 (691461)	0.12 (69643)	
Married—Spouse Absent	1.20 (3811958)	1.09 (3007753)	2.01 (804205)		1.06 (2738041)	1.85 (1073917)	
Widowed	4.75 (15045338)	4.42 (12215517)	7.06 (2829821)		3.87 (9999714)	8.67 (5045624)	
Divorced	8.01 (25378301)	7.75 (21447910)	9.80 (3930391)		7.36 (19019071)	10.93 (6359230)	
Separated	1.52 (4800817)	1.32 (3653987)	2.86 (1146830)		1.21 (3130049)	2.87 (1670767)	
Never married	43.79 (138710664)	42.30 (117015711)	54.10 (21694953)		42.26 (109272775)	50.59 (29437889)	
Personal Factor Income, $	30414.77 (64963.73)	34123.90 (68100.55)	4827.02 (24315.12)	<.001	36108.99 (69953.40)	5112.83 (21089.54)	<.001
No Personal Factor Income	37.31	33.81	61.46	<.001	32.28	59.66	<.001
SPM Resources	74106.20 (73729.35)	82697.93 (74908.07)	14835.36 (14322.05)	<.001	87008.29 (75614.17)	16776.44 (12605.11)	<.001
Household Size	3.21 (1.70)	3.23 (1.66)	3.05 (1.89)	<.001	3.27 (1.66)	2.89 (1.82)	<.001
Number Under Age 18 in Household	1.02 (1.32)	1.01 (1.30)	1.08 (1.44)	<.001	1.01 (1.29)	1.06 (1.44)	0.005
Percent Under Age 18 in Household	0.23 (0.26)	0.22 (0.25)	0.24 (0.28)	<.001	0.22 (0.25)	0.24 (0.28)	<.001
Number Age 65 or Older in Household	0.34 (0.65)	0.34 (0.66)	0.34 (0.62)	0.774	0.33 (0.65)	0.37 (0.64)	<.001
Percent Age 65 or Older in Household	0.17 (0.34)	0.17 (0.34)	0.18 (0.35)	<.001	0.16 (0.33)	0.22 (0.38)	<.001
Number of Working-age Adults in Household	1.84 (1.12)	1.88 (1.12)	1.63 (1.12)	<.001	1.93 (1.12)	1.46 (1.04)	<.001
Percent of Working-age Adults in Household	0.61 (0.33)	0.61 (0.33)	0.58 (0.35)	<.001	0.62 (0.32)	0.55 (0.36)	<.001
Number of Paid Laborers in Household	1.52 (1.02)	1.62 (1.00)	0.79 (0.85)	<.001	1.68 (0.99)	0.77 (0.79)	<.001
Percent of Paid Laborers in Household	0.51 (0.34)	0.55 (0.33)	0.28 (0.32)	<.001	0.56 (0.32)	0.28 (0.33)	<.001
Number of Disabled Working-age Adults in Household	0.09 (0.33)	0.07 (0.29)	0.22 (0.51)	<.001	0.07 (0.28)	0.21 (0.49)	<.001
Percent of Disabled Working-age Adults in Household	0.04 (0.14)	0.03 (0.12)	0.09 (0.24)	<.001	0.02 (0.11)	0.10 (0.25)	<.001
Out of Pocket Healthcare Expenditures Per Household Member, $	2022.54 (2832.29)	2101.17 (2417.81)	1480.07 (4764.23)	<.001	2139.24 (2421.26)	1503.99 (4157.88)	<.001
Role				<.001			<.001
Child	22.58 (71522953)	22.38 (61924007)	23.94 (9598947)		22.32 (57721153)	23.72 (13801801)	
Older Adult	16.74 (53031626)	16.58 (45869024)	17.86 (7162602)		15.65 (40478135)	21.57 (12553491)	
Disabled	3.69 (11673462)	2.85 (7874685)	9.47 (3798777)		2.34 (6061502)	9.64 (5611960)	
Student	2.74 (8688644)	2.20 (6073795)	6.52 (2614850)		2.28 (5882771)	4.82 (2805873)	
Caregiver	4.03 (12756769)	3.45 (9556609)	7.98 (3200160)		3.39 (8756843)	6.87 (3999925)	
Unemployed	0.48 (1508477)	0.27 (742571)	1.91 (765906)		0.25 (641040)	1.49 (867437)	
Other Not In Labor Force	3.05 (9676682)	2.58 (7138196)	6.33 (2538486)		2.51 (6497900)	5.46 (3178783)	
Paid Laborer	46.69 (147908235)	49.69 (137483761)	25.99 (10424475)		51.26 (132535189)	26.42 (15373046)	

N and percentages weighted to be nationally-representative

P-values are from t-tests for continuous variables and chi-squared tests for categorical variables

40.1 million individuals (12.7% of the population) were categorized as experiencing poverty under the absolute threshold (i.e., their income was less than their SPM poverty threshold). 58.2 million individuals (18.4% of the population) were categorized as experiencing poverty under the relative threshold (i.e., their income was below 50% of median).

**Fig 1:**
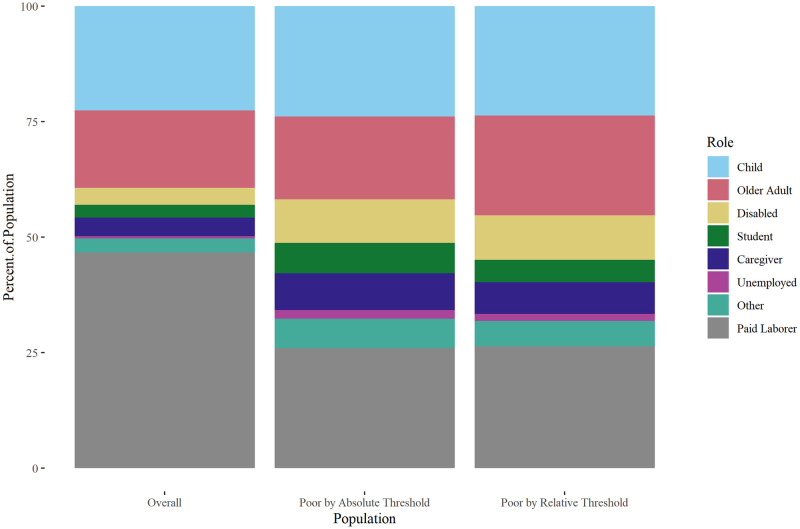
Distribution of roles, 2019 ASEC data. Distribution of roles in the overall U.S. population, and among those who experience poverty under the absolute and relative thresholds.

Risk of experiencing poverty was associated with roles (Fig 1). Compared with those in the paid labor force, those in all other roles were at increased risk of experiencing poverty under both the absolute and relative thresholds (Table 2). For example, the relative risk of experiencing poverty under the absolute threshold was 7.20 (95% CI 6.80 to 7.64, p < .001) for those in long-term unemployed roles, compared with those in the paid labor force. Similarly, the relative risk of experiencing poverty under the relative threshold was 1.86 (95% CI 1.80 to 1.91, p < .001) for children, compared with those in the paid labor force. Across the entire population, household composition was strongly associated with poverty risk. Having a greater percentage of household members be children, older adults, or disabled working-age adults were all associated with greater risk of poverty under the absolute and relative thresholds (p < .001 for all comparisons). Conversely, having an increasing percentage of the household consisting of those in the paid labor force was associated with lower risk of experiencing poverty under the absolute and relative thresholds (p < .001 for all comparisons).

**Table 2:** Poverty risk by role, 2019 ASEC data.

	Poverty by Absolute Threshold	Relative Risk	P
	% experiencing poverty (weighted N)	(95% CI)	
Overall	12.66 (40104203)	--	--
Child	13.42 (9598947)	1.90 (1.83 to 1.98)	<.001
Older Adult	13.51 (7162602)	1.92 (1.87 to 1.96)	<.001
Disabled	32.54 (3798777)	4.62 (4.48 to 4.75)	<.001
Student	30.10 (2614850)	4.27 (4.04 to 4.52)	<.001
Caregiver	25.09 (3200160)	3.56 (3.46 to 3.66)	<.001
Unemployed	50.77 (765906)	7.20 (6.80 to 7.64)	<.001
Other Not In Labor Force	26.23 (2538486)	3.72 (3.59 to 3.86)	<.001
Paid Laborer	7.05 (10424475)	ref	ref
	Poverty by Relative Threshold	Relative Risk	
	% experiencing poverty (weighted N)	(95% CI)	
Overall	18.37 (58192316)	--	--
Child	19.30 (13801801)	1.86 (1.80 to 1.91)	<.001
Older Adult	23.67 (12553491)	2.28 (2.22 to 2.34)	<.001
Disabled	48.07 (5611960)	4.63 (4.52 to 4.73)	<.001
Student	32.29 (2805873)	3.11 (2.94 to 3.28)	<.001
Caregiver	31.36 (3999925)	3.02 (2.94 to 3.10)	<.001
Unemployed	57.50 (867437)	5.53 (5.31 to 5.77)	<.001
Other Not In Labor Force	32.85 (3178783)	3.16 (3.06 to 3.26)	<.001
Paid Laborer	10.39 (15373046)	ref	ref

N and percent weighted to be nationally-representative.

Relative risk represents risk of experiencing poverty by a given threshold, compared with the risk observed for paid laborers

p-values are from predictive margins using delta-method standard errors are fitting a logistic regression model

Out-of-pocket healthcare expenditures were substantial, with mean $2022 (SD: $2832) in out-of-pocket spending per person.

### Experiences of poverty by role

#### Children

9.6 million children (13.4% of children) experienced poverty under the absolute threshold, and 13.8 million (19.3% of children) experienced poverty under the relative threshold. As children do not receive factor income from labor, they are dependent on intra-household transfers or transfer income from income support policies. Household composition is strongly associated with children’s poverty risk (**S1 Table in** S1 File). More children and more disabled working-age adults in the household are associated with experiencing poverty under both the absolute and relative thresholds, and more household members in the paid labor force is associated with lower risk (p < .001 for all comparisons). However, 72.0% of children in poverty under the absolute threshold and 78.0% of children in poverty under the relative threshold live in a household where at least one member is a paid laborer. The U.S. has relatively modest child benefits, with mean benefits of $888 (SD: $1,355) per child (**S2 Table in** S1 File).

#### Older adults

7.2 million older adults (13.5% of older adults) experienced poverty under the absolute threshold, and 12.6 million (23.7% of older adults) experienced poverty under the absolute threshold. 23.1% of older adults were still engaging in paid labor, and they were less likely to experience poverty under both the absolute (p < .001) and relative (p < .001) thresholds (**S3 Table in** S1 File). The U.S. system of benefits for older adults places heavy emphasis on (typically tax advantaged) private forms of older age benefits, such as private defined benefit and defined contribution retirement plans. 52.7% of those who report having only Social Security retirement income experience poverty under the absolute threshold, and 59.9% of those who report having only Social Security retirement income experience poverty under the relative threshold. This compares with poverty rates of 6.9% under the absolute threshold and 9.9% under the relative threshold for individuals who report Social Security retirement income along with defined benefit income (p < .001 for both comparisons), rates of 5.0% and 5.5% for individuals who report Social Security retirement income along with defined contribution income (p < .001 for both comparisons); and rates of 1.7% and 1.9% for those who report all three types of retirement income (p < .001 for both comparisons).

Household composition among older adults was again associated with poverty risk, with more children and disabled working-age adults associated with greater risk of poverty, and more household members in the paid labor force associated with lower risk of poverty, under both the absolute and relative thresholds (p< .001 for all comparisons).

Out-of-pocket healthcare spending was a major drain on resources, with mean spending of $3,453 (SD: $3,865) per household member.

#### Disabled individuals

3.8 million disabled working-age adults (32.5% of disabled working-age adults) experienced poverty under the absolute threshold, and 5.6 million (48.1%) experienced poverty under the relative threshold. Only 67.8% of individuals who reported not engaging in paid labor owing to disability reported receiving any form disability income (**S4 Table in** S1 File), suggesting a gap in reach of the disability insurance system. Further, disability income supports, when received, were modest, as the mean amount of disability income received from all sources (if any disability income was received) was $14,475 (SD: $10,937).

#### Students

2.6 million students (30.1% of students) experienced poverty under the absolute threshold, and 2.8 million (32.3%) experienced poverty under the relative threshold. Only 18.2% of students reported receiving income tied to the pursuit of education, and the mean amount among those who received any was $10,274 (SD: $13,583) (**S5 Table in** S1 File).

#### Caregivers

3.2 million caregivers (25.1% of caregivers) experienced poverty under the absolute threshold, and 4.0 million (31.4%) experienced poverty under the relative threshold. Only 1.9% of caregivers reported receiving income from programs historically meant for caregivers, such as TANF (Temporary Assistance to Needy Families) and other ‘cash welfare’ programs. Mean cash welfare income reported was $4,053 (SD: $3,361) if any was received. Household composition was again associated with poverty under both absolute and relative thresholds. Interestingly, more children in the household were not associated with greater poverty risk, but increasing percentage of older adults (p = .009 for poverty under absolute threshold and p = .006 for poverty under relative threshold) or disabled adults (p < .001 for poverty under both absolute threshold relative thresholds) were (**S6 Table in** S1 File).

#### Long-term unemployed individuals

766 thousand individuals unemployed for over one year (50.8% of long-term unemployed individuals) experienced poverty under the absolute threshold, and 867 thousand (57.5%) experienced poverty under the relative threshold. For people in this situation, unemployment insurance benefits are both rare and meager: only 6.8% of long-term unemployed individuals reported receiving any unemployment income, and the mean amount of unemployment income received, if any unemployment income was received, was $5,620 (SD: $4,278) (**S7 Table in** S1 File).

#### Other

2.5 million working-age individuals who report reasons not previously discussed for not engaging in paid labor (26.3% of the category) experienced poverty under the absolute threshold, and 3.2 million (32.9%) experienced poverty under the relative threshold. The principle reason given for not being in the paid labor force was early retirement (75.3%) (**S8 Table in** S1 File). Given the heterogeneous reasons for not engaging in paid labor in this sub-population, there is no specific set of programs meant to provide income support for individuals in this situation. Poverty risk was associated with fewer paid laborers in the household, under both the absolute (p < .001) and relative (p < .001) thresholds.

#### Paid laborers

Relative to their share of the population, paid laborers were less likely to experience poverty, but because they are a large segment of the population, paid laborers still represent the plurality of those who experience poverty under the absolute (10.4 million, 7.1% of paid laborers) and relative (15.4 million, 10.4% of paid laborers) thresholds.

88.0% of paid laborers received personal factor income greater than the absolute poverty threshold for a 1-person household, and 83.8% of paid laborers received factor income greater than the relative poverty threshold for a 1-person household (**S9 Table in** S1 File). This suggests that household composition is a key aspect of poverty risk for those who engage in paid labor. Specifically, under both the absolute and relative thresholds, poverty risk is greater with more children and disabled adults in the household (p < .001 for all comparisons).

Three other important reasons that paid laborers experience poverty are temporary unemployment, being out of work owing to personal poor health, and caregiving. 15.5% of those who experienced poverty under the absolute threshold had a spell of unemployment in the past year (compared with 5.4% of those who did not experience poverty under the absolute threshold, p < .001), and 14.2% of those who experienced poverty under the relative threshold had a spell of unemployment in the past year (compared with 4.2% of those who did not experience poverty under the relative threshold, p < .001). Only 16.1% of those who experienced unemployment reported receiving unemployment insurance income.

Of those who experienced poverty under the absolute threshold, 5.2% reported leaving a job owing to poor health, and 5.3% reported leaving a job for caregiving (compared with 2.3% and 1.7%, respectively, for those who did not experience poverty under the absolute threshold). Of those who experienced poverty under the relative threshold, 5.0% reported leaving a job owing to poor health, and 5.5% reported leaving a job for caregiving (compared with 1.9% and 2.4%, respectively, for those who did not experience poverty under the relative threshold). Only 29.3% of those who reported leaving a job for poor health reported receiving any disability income.

Finally, out-of-pocket medical expenditures were again a major drain on resources, with mean annual expenditures of $2,017 (SD: $2,646) per person.

### 2022 ASEC results

**Fig 2:**
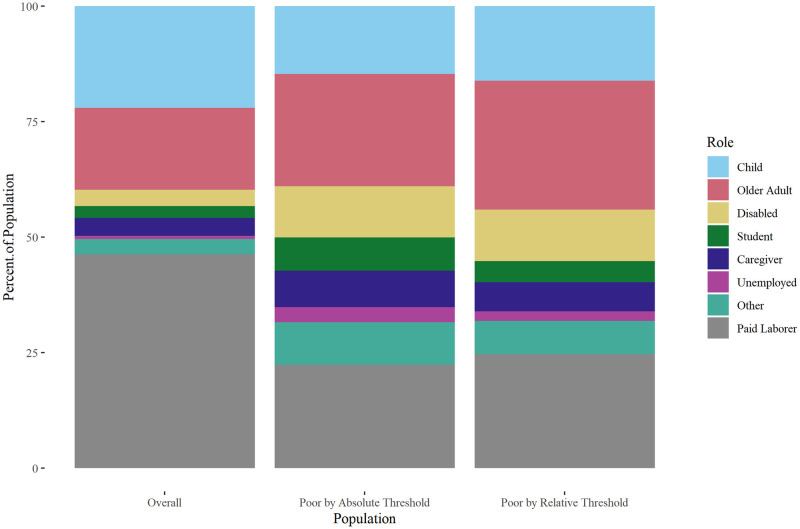
Distribution of roles, 2022 ASEC data. Distribution of roles in the overall U.S. population, and among those who experience poverty under the absolute and relative thresholds.

Results using 2022 ASEC data (covering the year 2021) were notable for strikingly lower poverty under the absolute and relative thresholds, compared with 2019 ASEC data (Fig 2 **and S10-S12 Tables in** S1 File). This was apparent both overall and among children, likely reflecting the impact of the expanded Child Tax Credit, along with other COVID-19 related income supports such as SNAP emergency allotments that were still active during this timeframe. Other than this however, the patterns observed regarding the association between role, household composition, and poverty risk were not meaningfully different from the analyses using 2019 ASEC data.

## Discussion

In this examination of nationally-representative data, we found empiric support for a structural view of poverty grounded in the centrality of markets as distributive institutions in the U.S [23, 26, 27, 49]. The roles one inhabits with relation to the market distribution of factor payments are strongly associated with poverty risk. Children and older adults are at substantially higher poverty risk than those engaging in paid labor—as are those who are disabled, students, caregivers, and those who are unemployed. Roles are not the entire story, however, given the aggregation of individuals into households. Even those who may have sufficient earnings to avoid poverty were they only supporting themselves see increased poverty risk with more household members who do not engage in paid labor, such as children or disabled working-age adults. In a market economy such as the U.S., the principle individual-level determinant of poverty risk is role, the principle household-level determinant is household composition, and the principle structural determinant is income support policy.

The approach used in this study has clear implications for social policy [49]. Past research has already found that socioecological factors, which include income support policies, are contributors to human life expectancy and the probability of surviving to age 100 or greater [7]. The framework used in this study may be useful for examining different parts of the life course that can differentially benefit from income support policies.

To avoid poverty, a household’s total income (factor income plus transfer income minus tax liabilities) must be greater than whatever poverty threshold is used. This means that the three general options for distributive institutions that could allow everyone to have sufficient income are a strategy that works through the factor payment system, a strategy that provides transfer income through income support policies, and a strategy that works via tax liabilities.

Low-income households typically have low tax liability at present, meaning that there is likely little room for this strategy to be effective. The factor income strategy typically involves trying to increase the amount of income received from paid labor. This strategy faces an initial problem in that about half the population of the U.S. does not earn factor income from labor for good reason (e.g., because they are older adults, children, or disabled). However, because many individuals pool resources through households, the factor income strategy might still work as those individuals who do not engage in paid labor could receive intra-household transfer income from those household members who receive factor income. Indeed, such an arrangement is common within many U.S. households. However, this strategy has limits. First, many individuals who do not engage in paid labor do not live in households with individuals who do. Second, the amount of factor income from labor received is typically determined by how that labor is valued in the market, not by the number of individuals being supported. In other words, a job that pays $75,000 a year will pay that amount whether the worker holding that job lives in a one-person household or a four-person household. However, a one-person household with $75,000 per year in factor income will have a very different standard of living than a four-person household with $75,000 per year in factor income. This suggests that the only way to ensure that sufficient income is distributed to all individuals regardless of household size, and regardless of the roles each person fills, is to have a comprehensive system of income support policies that distribute income to people who are unable to work. By helping to ensure that transfer income is distributed to those who cannot engage in paid labor, such distributive institutions have the effect of reducing poverty, even though their rationale is not to provide income to those who are ‘in need’ (that is, already poor) but rather to get transfer income to those who do not receive factor income.

Comparing the results of the analyses using 2022 and 2019 ASEC data reveals that income support policies enacted in relation to the COVID-19 pandemic may have affected poverty risk in the U.S. For example, we estimated that using 2019 data 13% of individuals experienced poverty under the absolute threshold and 18% experienced poverty under the relative threshold, while using 2022 data the corresponding estimates were 8% and 15%. These findings are consistent with other research that examined the effect of pandemic-era policies, including stimulus checks, enhanced unemployment insurance, the expanded child tax credit, and SNAP emergency allotments, and concluded that they reduced poverty [37, 50–53].

The findings of this study suggest directions for future research. Strengthening income support policies in each of the five areas emphasized—child benefits, older-age pensions, disability and sickness insurance, unemployment insurance, and out-of-pocket spending for healthcare—is likely to improve population health. This is true not only for those inhabiting roles directly affected, but also for those who are in households with people who would benefit. However, how best to strengthen income support policies in these areas remains an open question. Currently, much of the work examining health impacts of social policy is framed as examining whether some form of social policy in a particular area is better than none. However, moving forward, comparative effectiveness work that examines the comprehensive effects on all household members and costs of different approaches is likely to be more informative. Such work can draw on both international comparisons and state-level policy variation within the U.S.

The findings of this study should be interpreted in light of several limitations. First, this study used a cross-sectional design, and cannot establish a causal relationship between exposures and outcomes studied. Second, CPS data are self-reported. Income amounts, particularly at lower ranges, and participation in means-tested programs are known to be underreported [54]. Further, participants may not recall from which program they received income. However, the U.S. Census has developed a robust set of practices meant to mitigate this to the extent possible [54]. Moreover, it is unclear whether these issues of measurement error would affect the overall conclusions of the study—the findings that roles and household composition are strongly associated with poverty risk are likely to be robust to these issues. Third, this study put relatively little focus on policies that configure income distribution for those engaging in paid labor, as the overwhelming majority of such individuals receive enough factor income to avoid poverty if they had only themselves to support. However, this empirical observation reflects a specific policy context, not a universal truth. Current policies that produce this outcome are both the result of historical contestation over distributive institutions, and are always vulnerable to revanchist interests. Moreover, the results observed here with regard to those engaging in paid labor do not necessarily imply that the current factor payment system is just, or is not also in need of reform. Finally, though the supplemental poverty measure income concept is clearly preferable to the Official Poverty Measure income concept, it does have its own limitations [46].

## Conclusions

A market-based factor payment system distributes income unequally, based on the roles individuals inhabit. Further, household composition is heterogeneous, with respect to the roles its members inhabit. For these reasons, any society that uses markets as primary distributive institutions will distribute income unequally, with poverty as an extreme manifestation, unless the factor payment system is complemented by a strong system of income support policies.

Fundamentally, society must provide the power to consume the products its members need to maintain health. Some products, like basic education, are allocated publically, under administrative mechanisms. But many other products are allocated by the market. Income provides purchasing power that can become consumption power for goods and services allocated by market mechanisms. For those who do not receive income through the factor payment system, there must be some other way of providing income. Understanding the issues of poverty, and income distribution more broadly, in this way helps make clear the inescapable role of strong income support policies for improving income distribution and reducing poverty risk.

## Supporting information

S1 File Online supplemental content for: Gaps in the welfare state: A role-based model of poverty risk in the U.S. (DOCX)
