# Supplementary material for: Gaps in the welfare state: A role-based model of poverty risk in the U.S
Source: PLoS One. 2023 Apr 13;18(4):e0284251. doi: 10.1371/journal.pone.0284251 (PMC10101477; doi:10.1371/journal.pone.0284251)
Supplement: S1 File — (DOCX) [file pone.0284251.s001.docx]

Online Supplemental Content for:

**Gaps in the Welfare State: A Role-Based Model of Poverty Risk in the U.S.**

***Technical Appendix***

*Variable Construction*

This section details definitions and constructions of key variables used in the manuscript. Personal factor income consists of all income from labor earnings (e.g., wages and self-employment income) and capital holdings (e.g., capital gains, dividends, interest, and rental income). Value of child benefits, per child, was calculated as the sum of earned income tax credit (EITC) and the additional child tax credit (the refundable portion of the child tax credit) received by the household, divided by the number of children (age < 18 years). Income from defined benefit retirement plans was calculated as the sum of income from annuities, pensions, survival benefits, and Veteran’s Affairs retirement benefits. Income from defined contribution retirement plans was calculated as the sum of income from distributions and interest of retirement accounts. Total retirement income was calculated as the sum of personal income from defined benefit plans, defined contribution plans, and social security OASI (Old Age and Survivor’s Insurance). Supplemental security income is reported separately from retirement income as it is not specifically a retirement benefit. The indicator variable “Individual Social Security Income Above Absolute Poverty Threshold” was calculated by comparing an individual’s social security OASI income and comparing it to the median SPM resources poverty threshold for a 1 person household. For 2019 ASEC data, this amount was $12,579. This variable helps approximate whether, if an individual had only Social Security OASI income and needed to support only themselves with it, they would be above the absolute poverty threshold. Similarly, the indicator variable “Individual Social Security Income Above Relative Poverty Threshold” was calculated by comparing an individual’s Social Security OASI income and comparing it to 50% of national median SPM resources in a given year. For 2019 ASEC data, this amount was $16,471.50. This variable helps approximate whether, if an individual had only Social Security OASI income and needed to support only themselves with it, they would be above the relative poverty threshold. Total disability income was calculated as the sum of income from Supplemental Security Income (SSI), Social Security Disability Insurance (SSDI) income, worker’s compensation programs, Veteran’s Affairs disability benefits, and other (typically private) disability sources. Financial assistance income is income received from family and friends for financial assistance. The indicator variable “Individual Personal Income Above Absolute Poverty Threshold” was calculated by comparing an individual’s personal factor income and comparing it to the median SPM resources poverty threshold for a 1 person household. For 2019 ASEC data, this amount was $12,579. This variable helps approximate whether, if an individual had only their personal factor income and needed to support only themselves with it, they would be above the absolute poverty threshold. Similarly, the indicator variable “Individual Personal Income Above Relative Poverty Threshold” was calculated by comparing an individual’s personal factor income and comparing it to 50% of national median SPM resources in a given year. For 2019 ASEC data, this amount was $16,471.50. This variable helps approximate whether, if an individual had only their personal factor income and needed to support only themselves with it, they would be above the relative poverty threshold.

| Table S1: Demographics by Role, 2019 ASEC Data | | | | | | | |
| --- | --- | --- | --- | --- | --- | --- | --- |
| Child | | | | | | | |
|  | Overall | Poverty Under Absolute Threshold | | | Poverty Under Relative Threshold | | |
|  |  | Not in Poverty | In Poverty | p-value | Not in Poverty | In Poverty | p-value |
| Weighted N | 71522953 | 61924007 | 9598947 |  | 57721153 | 13801801 |  |
|  | % or mean (SD) | % or mean (SD) | % or mean (SD) |  | % or mean (SD) | % or mean (SD) |  |
| Age, y | 8.78 (5.11) | 8.80 (5.11) | 8.69 (5.13) | 0.431 | 8.85 (5.11) | 8.50 (5.08) | <.001 |
| Female | 48.86 | 49.02 | 47.80 | 0.003 | 49.14 | 47.67 | <.001 |
| Race and Ethnicity | |  |  | <.001 |  |  | <.001 |
| Non-Hispanic White | 51.17 | 54.91 | 27.01 |  | 55.73 | 32.10 |  |
| Non-Hispanic Black | 12.89 | 11.38 | 22.67 |  | 10.70 | 22.05 |  |
| Non-Hispanic American Indian and Alaskan Native | 0.96 | 0.92 | 1.21 |  | 0.83 | 1.51 |  |
| Non-Hispanic Asian | 6.11 | 6.23 | 5.36 |  | 6.58 | 4.16 |  |
| Non-Hispanic Native Hawaiian and Other Pacific Islander | 0.33 | 0.32 | 0.44 |  | 0.31 | 0.42 |  |
| Non-Hispanic Multi-Racial | 3.96 | 4.02 | 3.54 |  | 4.02 | 3.67 |  |
| Hispanic, any race | 24.58 | 22.22 | 39.78 |  | 21.82 | 36.09 |  |
| Education | |  |  | -- |  |  | -- |
| Child, education incomplete | -- | -- | -- |  | -- | -- |  |
| Less than HS Diploma | -- | -- | -- |  | -- | -- |  |
| HS Diploma or GED | -- | -- | -- |  | -- | -- |  |
| Greater than HS Diploma | -- | -- | -- |  | -- | -- |  |
| Marital Status | |  |  | 0.125 |  |  | 0.043 |
| Married - Civilian Spouse Present | 0.05 | 0.06 | 0.05 |  | 0.06 | 0.03 |  |
| Married - Armed Forces Spouse Present | 0.00 | 0.00 | 0.00 |  | 0.00 | 0.00 |  |
| Married - Spouse Absent | 0.11 | 0.12 | 0.09 |  | 0.11 | 0.11 |  |
| Widowed | 0.02 | 0.02 | 0.03 |  | 0.02 | 0.02 |  |
| Divorced | 0.06 | 0.06 | 0.03 |  | 0.06 | 0.03 |  |
| Separated | 0.09 | 0.08 | 0.16 |  | 0.08 | 0.13 |  |
| Never married | 99.67 | 99.68 | 99.64 |  | 99.67 | 99.68 |  |
| Personal Factor Income, $ | 274.08 (3624.04) | 306.92 (3883.35) | 62.25 (723.66) | <.001 | 325.13 (4018.13) | 60.61 (694.55) | <.001 |
| No Personal Factor Income | 95.40 | 95.03 | 97.83 | <.001 | 94.83 | 97.79 | <.001 |
| SPM Resources, $ | 78926.27 (72147.80) | 87949.73 (73345.30) | 20714.84 (12943.81) | <.001 | 92239.58 (74157.43) | 23248.06 (11592.66) | <.001 |
| Household Size | 4.52 (1.48) | 4.53 (1.44) | 4.52 (1.72) | 0.889 | 4.54 (1.44) | 4.46 (1.64) | 0.013 |
| Number Under Age 18 in Household | 2.40 (1.22) | 2.37 (1.20) | 2.57 (1.34) | <.001 | 2.34 (1.19) | 2.61 (1.33) | <.001 |
| Percent Under Age 18 in Household | 0.52 (0.14) | 0.51 (0.14) | 0.56 (0.16) | <.001 | 0.50 (0.14) | 0.57 (0.15) | <.001 |
| Number Age 65 or Older in Household | 0.08 (0.32) | 0.07 (0.32) | 0.10 (0.34) | <.001 | 0.08 (0.32) | 0.08 (0.32) | 0.205 |
| Percent Age 65 or Older in Household | 0.02 (0.07) | 0.02 (0.07) | 0.02 (0.08) | <.001 | 0.02 (0.07) | 0.02 (0.08) | 0.001 |
| Number of Working Age Adults in Household | 2.05 (0.78) | 2.08 (0.76) | 1.85 (0.91) | <.001 | 2.12 (0.76) | 1.78 (0.82) | <.001 |
| Percent of Working Age Adults in Household | 0.47 (0.15) | 0.48 (0.14) | 0.42 (0.16) | <.001 | 0.48 (0.14) | 0.41 (0.16) | <.001 |
| Number of Paid Laborers in Household | 1.67 (0.84) | 1.78 (0.79) | 0.98 (0.81) | <.001 | 1.83 (0.79) | 1.04 (0.75) | <.001 |
| Percent of Paid Laborers in Household | 0.39 (0.19) | 0.41 (0.18) | 0.22 (0.19) | <.001 | 0.42 (0.18) | 0.24 (0.18) | <.001 |
| Number of Disabled Working Age Adults in Household | 0.06 (0.28) | 0.05 (0.23) | 0.18 (0.45) | <.001 | 0.04 (0.22) | 0.16 (0.43) | <.001 |
| Percent of Disabled Working Age Adults in Household | 0.01 (0.07) | 0.01 (0.05) | 0.04 (0.11) | <.001 | 0.01 (0.05) | 0.04 (0.11) | <.001 |
| Out of Pocket Healthcare Expenditures Per Household Member, $ | 1256.89 (1889.83) | 1325.35 (1511.78) | 815.23 (3412.30) | <.001 | 1364.22 (1511.17) | 808.01 (2951.00) | <.001 |
|  |  |  |  |  |  |  |  |
| Older Adult | | | | | | | |
|  | Overall | Poverty Under Absolute Threshold | | | Poverty Under Relative Threshold | | |
|  |  | Not in Poverty | In Poverty | p-value | Not in Poverty | In Poverty | p-value |
| Weighted N | 53031626 | 45869024 | 7162602 |  | 40478135 | 12553491 |  |
|  | % or mean (SD) | % or mean (SD) | % or mean (SD) |  | % or mean (SD) | % or mean (SD) |  |
| Age, y | 73.51 (6.16) | 73.38 (6.09) | 74.39 (6.53) | <.001 | 73.14 (6.00) | 74.71 (6.52) | <.001 |
| Female | 54.61 | 53.72 | 60.28 | <.001 | 52.81 | 60.42 | <.001 |
| Race and Ethnicity | |  |  | <.001 |  |  | <.001 |
| Non-Hispanic White | 76.17 | 78.70 | 59.93 |  | 79.14 | 66.57 |  |
| Non-Hispanic Black | 9.22 | 8.33 | 14.93 |  | 7.81 | 13.75 |  |
| Non-Hispanic American Indian and Alaskan Native | 0.54 | 0.48 | 0.95 |  | 0.40 | 0.98 |  |
| Non-Hispanic Asian | 4.59 | 4.23 | 6.88 |  | 4.60 | 4.56 |  |
| Non-Hispanic Native Hawaiian and Other Pacific Islander | 0.16 | 0.14 | 0.28 |  | 0.15 | 0.19 |  |
| Non-Hispanic Multi-Racial | 0.79 | 0.78 | 0.85 |  | 0.78 | 0.82 |  |
| Hispanic, any race | 8.53 | 7.34 | 16.17 |  | 7.11 | 13.13 |  |
| Education | |  |  | <.001 |  |  | <.001 |
| Child, education incomplete | 0.00 | 0.00 | 0.00 |  | 0.00 | 0.00 |  |
| Less than HS Diploma | 12.28 | 10.32 | 24.89 |  | 8.67 | 23.93 |  |
| HS Diploma or GED | 31.35 | 30.82 | 34.79 |  | 29.47 | 37.41 |  |
| Greater than HS Diploma | 56.36 | 58.87 | 40.33 |  | 61.85 | 38.66 |  |
| Marital Status | |  |  | <.001 |  |  | <.001 |
| Married - Civilian Spouse Present | 56.85 | 59.37 | 40.69 |  | 62.27 | 39.37 |  |
| Married - Armed Forces Spouse Present | 0 | 0.00 | 0.00 |  | 0.00 | 0.00 |  |
| Married - Spouse Absent | 1.44 | 1.32 | 2.24 |  | 1.27 | 2.01 |  |
| Widowed | 21.94 | 20.85 | 28.93 |  | 19.06 | 31.23 |  |
| Divorced | 12.83 | 12.15 | 17.21 |  | 11.43 | 17.34 |  |
| Separated | 1.23 | 1.06 | 2.32 |  | 0.98 | 2.02 |  |
| Never married | 5.7 | 5.25 | 8.60 |  | 4.98 | 8.02 |  |
| Personal Factor Income, $ | 15933.67 (51981.97) | 18056.30 (54340.69) | 2340.45 (29711.77) | <.001 | 20272.87 (57403.19) | 1942.14 (23102.62) | <.001 |
| No Personal Factor Income | 34.34 | 29.94 | 62.49 | <.001 | 26.32 | 60.20 | <.001 |
| SPM Resources, $ | 57930.13 (61426.08) | 65497.97 (62541.83) | 9466.00 (13112.38) | <.001 | 71987.88 (63819.12) | 12601.58 (10720.05) | <.001 |
| Household Size | 2.02 (1.08) | 2.04 (1.07) | 1.89 (1.15) | <.001 | 2.12 (1.10) | 1.73 (0.96) | <.001 |
| Number Under Age 18 in Household | 0.10 (0.46) | 0.10 (0.45) | 0.13 (0.53) | <.001 | 0.11 (0.47) | 0.09 (0.44) | 0.008 |
| Percent Under Age 18 in Household | 0.02 (0.09) | 0.02 (0.09) | 0.03 (0.11) | <.001 | 0.02 (0.09) | 0.02 (0.09) | <.001 |
| Number Age 65 or Older in Household | 1.51 (0.52) | 1.54 (0.52) | 1.38 (0.51) | <.001 | 1.56 (0.52) | 1.37 (0.50) | <.001 |
| Percent Age 65 or Older in Household | 0.85 (0.26) | 0.84 (0.26) | 0.85 (0.26) | 0.562 | 0.83 (0.26) | 0.88 (0.23) | <.001 |
| Number of Working Age Adults in Household | 0.40 (0.78) | 0.41 (0.79) | 0.38 (0.75) | 0.005 | 0.45 (0.83) | 0.27 (0.61) | <.001 |
| Percent of Working Age Adults in Household | 0.13 (0.23) | 0.13 (0.23) | 0.12 (0.22) | 0.001 | 0.14 (0.23) | 0.10 (0.20) | <.001 |
| Number of Paid Laborers in Household | 0.62 (0.86) | 0.67 (0.88) | 0.24 (0.54) | <.001 | 0.74 (0.91) | 0.20 (0.47) | <.001 |
| Percent of Paid Laborers in Household | 0.27 (0.36) | 0.30 (0.37) | 0.10 (0.24) | <.001 | 0.33 (0.38) | 0.10 (0.24) | <.001 |
| Number of Disabled Working Age Adults in Household | 0.04 (0.22) | 0.04 (0.20) | 0.09 (0.33) | <.001 | 0.04 (0.20) | 0.07 (0.29) | <.001 |
| Percent of Disabled Working Age Adults in Household | 0.02 (0.08) | 0.01 (0.07) | 0.03 (0.11) | <.001 | 0.01 (0.07) | 0.02 (0.10) | <.001 |
| Out of Pocket Healthcare Expenditures Per Household Member, $ | 3453.49 (3865.31) | 3569.15 (3064.94) | 2712.76 (7059.72) | <.001 | 3702.00 (3079.47) | 2652.18 (5630.36) | <.001 |
|  |  |  |  |  |  |  |  |
| Disabled | | | | | | | |
|  | Overall | Poverty Under Absolute Threshold | | | Poverty Under Relative Threshold | | |
|  |  | Not in Poverty | In Poverty | p-value | Not in Poverty | In Poverty | p-value |
| Weighted N | 11673462 | 7874685 | 3798777 |  | 6061502 | 5611960 |  |
|  | % or mean (SD) | % or mean (SD) | % or mean (SD) |  | % or mean (SD) | % or mean (SD) |  |
| Age, y | 49.00 (12.25) | 49.34 (12.42) | 48.31 (11.87) | <.001 | 48.63 (12.72) | 49.40 (11.71) | 0.031 |
| Female | 52.98 | 53.48 | 51.94 | 0.044 | 53.13 | 52.81 | 0.726 |
| Race and Ethnicity | |  |  | <.001 |  |  | <.001 |
| Non-Hispanic White | 59.34 | 62.47 | 52.86 |  | 62.67 | 55.75 |  |
| Non-Hispanic Black | 20.72 | 18.37 | 25.58 |  | 16.80 | 24.95 |  |
| Non-Hispanic American Indian and Alaskan Native | 1.74 | 1.58 | 2.06 |  | 1.41 | 2.10 |  |
| Non-Hispanic Asian | 2.19 | 2.20 | 2.15 |  | 2.56 | 1.78 |  |
| Non-Hispanic Native Hawaiian and Other Pacific Islander | 0.1 | 0.13 | 0.03 |  | 0.13 | 0.07 |  |
| Non-Hispanic Multi-Racial | 2.09 | 2.25 | 1.75 |  | 2.19 | 1.98 |  |
| Hispanic, any race | 13.82 | 12.99 | 15.56 |  | 14.24 | 13.38 |  |
| Education | |  |  | <.001 |  |  | <.001 |
| Child, education incomplete | 0.00 | 0.00 | 0.00 |  | 0.00 | 0.00 |  |
| Less than HS Diploma | 24 | 20.64 | 30.95 |  | 19.15 | 29.23 |  |
| HS Diploma or GED | 42.59 | 43.33 | 41.07 |  | 43.78 | 41.31 |  |
| Greater than HS Diploma | 33.41 | 36.03 | 27.98 |  | 37.07 | 29.46 |  |
| Marital Status | |  |  | <.001 |  |  | <.001 |
| Married - Civilian Spouse Present | 30.96 | 36.11 | 20.27 |  | 40.66 | 20.47 |  |
| Married - Armed Forces Spouse Present | 0.09 | 0.14 | 0.00 |  | 0.18 | 0.00 |  |
| Married - Spouse Absent | 1.42 | 1.07 | 2.14 |  | 0.94 | 1.95 |  |
| Widowed | 5.64 | 5.35 | 6.23 |  | 4.18 | 7.21 |  |
| Divorced | 21.27 | 20.35 | 23.18 |  | 17.64 | 25.19 |  |
| Separated | 4.72 | 4.17 | 5.85 |  | 3.32 | 6.23 |  |
| Never married | 35.9 | 32.81 | 42.33 |  | 33.08 | 38.96 |  |
| Personal Factor Income, $ | 243.89 (4526.57) | 338.32 (5457.93) | 48.13 (1077.54) | <.001 | 432.14 (6212.76) | 40.56 (925.79) | <.001 |
| No Personal Factor Income | 82.47 | 79.11 | 89.44 | <.001 | 76.98 | 88.40 | <.001 |
| SPM Resources, $ | 36427.21 (41273.64) | 48417.45 (45169.58) | 11572.01 (9475.41) | <.001 | 57641.59 (47683.77) | 13513.47 (8756.27) | <.001 |
| Household Size | 2.53 (1.52) | 2.62 (1.47) | 2.34 (1.59) | <.001 | 2.87 (1.48) | 2.16 (1.47) | <.001 |
| Number Under Age 18 in Household | 0.40 (0.88) | 0.37 (0.83) | 0.46 (0.98) | <.001 | 0.41 (0.86) | 0.39 (0.91) | 0.192 |
| Percent Under Age 18 in Household | 0.09 (0.18) | 0.08 (0.17) | 0.11 (0.21) | <.001 | 0.09 (0.17) | 0.09 (0.19) | 0.241 |
| Number Age 65 or Older in Household | 0.20 (0.48) | 0.22 (0.50) | 0.17 (0.43) | <.001 | 0.25 (0.53) | 0.15 (0.41) | <.001 |
| Percent Age 65 or Older in Household | 0.07 (0.17) | 0.08 (0.18) | 0.06 (0.15) | <.001 | 0.09 (0.19) | 0.06 (0.15) | <.001 |
| Number of Working Age Adults in Household | 1.93 (1.01) | 2.03 (1.02) | 1.71 (0.94) | <.001 | 2.21 (1.05) | 1.62 (0.86) | <.001 |
| Percent of Working Age Adults in Household | 0.84 (0.23) | 0.84 (0.23) | 0.83 (0.24) | 0.482 | 0.82 (0.23) | 0.85 (0.23) | <.001 |
| Number of Paid Laborers in Household | 0.61 (0.82) | 0.77 (0.88) | 0.26 (0.54) | <.001 | 0.96 (0.90) | 0.23 (0.49) | <.001 |
| Percent of Paid Laborers in Household | 0.19 (0.24) | 0.25 (0.25) | 0.08 (0.16) | <.001 | 0.30 (0.24) | 0.08 (0.17) | <.001 |
| Number of Disabled Working Age Adults in Household | 1.19 (0.47) | 1.16 (0.41) | 1.24 (0.57) | <.001 | 1.16 (0.41) | 1.21 (0.53) | <.001 |
| Percent of Disabled Working Age Adults in Household | 0.61 (0.30) | 0.57 (0.29) | 0.69 (0.31) | <.001 | 0.50 (0.26) | 0.72 (0.30) | <.001 |
| Out of Pocket Healthcare Expenditures Per Household Member, $ | 1414.47 (2526.46) | 1569.26 (2159.35) | 1093.60 (3130.52) | <.001 | 1768.13 (2256.38) | 1032.47 (2738.47) | <.001 |
|  |  |  |  |  |  |  |  |
| Student | | | | | | | |
|  | Overall | Poverty Under Absolute Threshold | | | Poverty Under Relative Threshold | | |
|  |  | Not in Poverty | In Poverty | p-value | Not in Poverty | In Poverty | p-value |
| Weighted N | 8688644 | 6073795 | 2614850 |  | 5882771 | 2805873 |  |
|  | % or mean (SD) | % or mean (SD) | % or mean (SD) |  | % or mean (SD) | % or mean (SD) |  |
| Age, y | 23.39 (7.57) | 22.81 (7.30) | 24.74 (7.99) | <.001 | 22.78 (7.30) | 24.68 (7.94) | <.001 |
| Female | 48.90 | 50.27 | 45.71 | <.001 | 50.35 | 45.86 | <.001 |
| Race and Ethnicity | |  |  | <.001 |  |  | <.001 |
| Non-Hispanic White | 45.96 | 49.2 | 38.43 |  | 48.64 | 40.33 |  |
| Non-Hispanic Black | 13.53 | 12.87 | 15.06 |  | 12.69 | 15.30 |  |
| Non-Hispanic American Indian and Alaskan Native | 0.92 | 0.96 | 0.84 |  | 0.75 | 1.28 |  |
| Non-Hispanic Asian | 13.13 | 10.96 | 18.18 |  | 11.14 | 17.32 |  |
| Non-Hispanic Native Hawaiian and Other Pacific Islander | 0.34 | 0.33 | 0.37 |  | 0.34 | 0.36 |  |
| Non-Hispanic Multi-Racial | 2.13 | 2.49 | 1.28 |  | 2.46 | 1.44 |  |
| Hispanic, any race | 23.98 | 23.19 | 25.83 |  | 23.99 | 23.97 |  |
| Education | |  |  | <.001 |  |  | <.001 |
| Child, education incomplete | 0.00 | 0.00 | 0.00 |  | 0.00 | 0.00 |  |
| Less than HS Diploma | 23.1 | 24.82 | 19.12 |  | 24.66 | 19.83 |  |
| HS Diploma or GED | 16.97 | 16.71 | 17.58 |  | 16.35 | 18.28 |  |
| Greater than HS Diploma | 59.92 | 58.47 | 63.30 |  | 58.98 | 61.90 |  |
| Marital Status | |  |  | <.001 |  |  | <.001 |
| Married - Civilian Spouse Present | 7.92 | 8.35 | 6.91 |  | 8.32 | 7.07 |  |
| Married - Armed Forces Spouse Present | 0.34 | 0.46 | 0.07 |  | 0.42 | 0.19 |  |
| Married - Spouse Absent | 1.1 | 0.84 | 1.70 |  | 0.85 | 1.62 |  |
| Widowed | 0.4 | 0.28 | 0.69 |  | 0.29 | 0.64 |  |
| Divorced | 1.41 | 1.01 | 2.34 |  | 0.90 | 2.47 |  |
| Separated | 0.86 | 0.61 | 1.45 |  | 0.62 | 1.36 |  |
| Never married | 87.97 | 88.46 | 86.84 |  | 88.60 | 86.65 |  |
| Personal Factor Income, $ | 123.81 (2290.62) | 149.90 (2646.73) | 63.22 (1076.81) | 0.003 | 152.89 (2688.79) | 62.84 (1042.36) | 0.003 |
| No Personal Factor Income | 84.21 | 83.30 | 86.29 | <.001 | 83.15 | 86.40 | <.001 |
| SPM Resources, $ | 66060.36 (77648.15) | 89334.87 (82034.05) | 11998.15 (14899.10) | <.001 | 91830.17 (82211.53) | 12031.59 (13741.72) | <.001 |
| Household Size | 3.59 (1.78) | 3.93 (1.61) | 2.80 (1.92) | <.001 | 3.99 (1.58) | 2.74 (1.87) | <.001 |
| Number Under Age 18 in Household | 0.68 (1.04) | 0.74 (1.03) | 0.56 (1.06) | <.001 | 0.73 (1.02) | 0.60 (1.08) | 0.001 |
| Percent Under Age 18 in Household | 0.14 (0.18) | 0.15 (0.18) | 0.12 (0.19) | <.001 | 0.14 (0.18) | 0.13 (0.19) | 0.013 |
| Number Age 65 or Older in Household | 0.13 (0.41) | 0.14 (0.42) | 0.10 (0.37) | 0.001 | 0.14 (0.43) | 0.09 (0.35) | <.001 |
| Percent Age 65 or Older in Household | 0.03 (0.11) | 0.03 (0.11) | 0.03 (0.10) | 0.024 | 0.03 (0.11) | 0.03 (0.10) | <.001 |
| Number of Working Age Adults in Household | 2.78 (1.24) | 3.05 (1.13) | 2.13 (1.26) | <.001 | 3.12 (1.12) | 2.05 (1.16) | <.001 |
| Percent of Working Age Adults in Household | 0.83 (0.20) | 0.82 (0.20) | 0.85 (0.21) | <.001 | 0.82 (0.19) | 0.85 (0.21) | 0.002 |
| Number of Paid Laborers in Household | 1.30 (1.03) | 1.63 (0.95) | 0.53 (0.76) | <.001 | 1.67 (0.94) | 0.51 (0.71) | <.001 |
| Percent of Paid Laborers in Household | 0.33 (0.24) | 0.41 (0.21) | 0.13 (0.19) | <.001 | 0.42 (0.21) | 0.14 (0.19) | <.001 |
| Number of Disabled Working Age Adults in Household | 0.07 (0.29) | 0.06 (0.26) | 0.09 (0.34) | 0.001 | 0.06 (0.26) | 0.10 (0.34) | <.001 |
| Percent of Disabled Working Age Adults in Household | 0.02 (0.08) | 0.02 (0.07) | 0.03 (0.09) | <.001 | 0.01 (0.06) | 0.03 (0.10) | <.001 |
| Out of Pocket Healthcare Expenditures Per Household Member, $ | 1357.35 (2257.95) | 1499.67 (1753.35) | 1026.76 (3106.32) | <.001 | 1508.74 (1712.35) | 1039.95 (3081.58) | <.001 |
|  |  |  |  |  |  |  |  |
| Caregiver | | | | | | | |
|  | Overall | Poverty Under Absolute Threshold | | | Poverty Under Relative Threshold | | |
|  |  | Not in Poverty | In Poverty | p-value | Not in Poverty | In Poverty | p-value |
| Weighted N | 12756769 | 9556609 | 3200160 |  | 8756843 | 3999925 |  |
|  | % or mean (SD) | % or mean (SD) | % or mean (SD) |  | % or mean (SD) | % or mean (SD) |  |
| Age, y | 40.92 (11.74) | 41.42 (11.60) | 39.43 (12.03) | <.001 | 41.73 (11.51) | 39.16 (12.05) | <.001 |
| Female | 91.48 | 92.89 | 87.29 | <.001 | 93.07 | 88.00 | <.001 |
| Race and Ethnicity | |  |  | <.001 |  |  | <.001 |
| Non-Hispanic White | 50.31 | 55.79 | 33.94 |  | 55.89 | 38.08 |  |
| Non-Hispanic Black | 8.58 | 5.72 | 17.14 |  | 5.62 | 15.07 |  |
| Non-Hispanic American Indian and Alaskan Native | 1.15 | 1.16 | 1.10 |  | 0.84 | 1.81 |  |
| Non-Hispanic Asian | 10.07 | 10.65 | 8.35 |  | 11.29 | 7.41 |  |
| Non-Hispanic Native Hawaiian and Other Pacific Islander | 0.4 | 0.36 | 0.54 |  | 0.39 | 0.42 |  |
| Non-Hispanic Multi-Racial | 1.08 | 1.13 | 0.93 |  | 1.02 | 1.19 |  |
| Hispanic, any race | 28.41 | 25.20 | 38.00 |  | 24.94 | 36.01 |  |
| Education | |  |  | <.001 |  |  | <.001 |
| Child, education incomplete | 0.00 | 0.00 | 0.00 |  | 0.00 | 0.00 |  |
| Less than HS Diploma | 18.2 | 15.03 | 27.65 |  | 14.40 | 26.50 |  |
| HS Diploma or GED | 31.84 | 29.68 | 38.28 |  | 28.69 | 38.72 |  |
| Greater than HS Diploma | 49.97 | 55.29 | 34.07 |  | 56.91 | 34.79 |  |
| Marital Status | |  |  | <.001 |  |  | <.001 |
| Married - Civilian Spouse Present | 72.35 | 81.85 | 43.97 |  | 83.23 | 48.52 |  |
| Married - Armed Forces Spouse Present | 1.78 | 2.21 | 0.49 |  | 2.10 | 1.08 |  |
| Married - Spouse Absent | 1.4 | 0.61 | 3.78 |  | 0.65 | 3.05 |  |
| Widowed | 1.54 | 1.20 | 2.53 |  | 1.11 | 2.48 |  |
| Divorced | 3.7 | 2.52 | 7.24 |  | 2.05 | 7.32 |  |
| Separated | 1.78 | 0.88 | 4.47 |  | 0.74 | 4.06 |  |
| Never married | 17.44 | 10.72 | 37.52 |  | 10.12 | 33.48 |  |
| Personal Factor Income, $ | 995.98 (12452.55) | 1211.84 (13890.95) | 351.38 (6433.89) | <.001 | 1309.93 (14500.10) | 308.67 (5796.34) | <.001 |
| No Personal Factor Income | 70.26 | 64.79 | 86.59 | <.001 | 63.03 | 86.07 | <.001 |
| SPM Resources, $ | 63651.55 (71671.00) | 79725.44 (75886.74) | 15650.26 (14275.17) | <.001 | 84765.18 (77343.27) | 17428.52 (13197.82) | <.001 |
| Household Size | 3.87 (1.64) | 3.97 (1.59) | 3.57 (1.74) | <.001 | 4.02 (1.60) | 3.55 (1.68) | <.001 |
| Number Under Age 18 in Household | 1.46 (1.38) | 1.49 (1.38) | 1.37 (1.37) | <.001 | 1.48 (1.38) | 1.40 (1.39) | <.001 |
| Percent Under Age 18 in Household | 0.31 (0.25) | 0.31 (0.24) | 0.31 (0.26) | 0.162 | 0.31 (0.24) | 0.31 (0.26) | 0.453 |
| Number Age 65 or Older in Household | 0.10 (0.33) | 0.09 (0.32) | 0.12 (0.36) | 0.001 | 0.10 (0.33) | 0.11 (0.33) | 0.119 |
| Percent Age 65 or Older in Household | 0.03 (0.11) | 0.03 (0.10) | 0.03 (0.11) | 0.009 | 0.03 (0.10) | 0.04 (0.12) | 0.006 |
| Number of Working Age Adults in Household | 2.32 (0.92) | 2.39 (0.89) | 2.09 (0.97) | <.001 | 2.44 (0.91) | 2.04 (0.87) | <.001 |
| Percent of Working Age Adults in Household | 0.66 (0.24) | 0.66 (0.24) | 0.66 (0.26) | 0.866 | 0.66 (0.23) | 0.65 (0.26) | 0.007 |
| Number of Paid Laborers in Household | 1.09 (0.76) | 1.24 (0.72) | 0.65 (0.71) | <.001 | 1.29 (0.73) | 0.67 (0.65) | <.001 |
| Percent of Paid Laborers in Household | 0.29 (0.19) | 0.33 (0.17) | 0.17 (0.18) | <.001 | 0.34 (0.16) | 0.18 (0.18) | <.001 |
| Number of Disabled Working Age Adults in Household | 0.06 (0.25) | 0.04 (0.20) | 0.11 (0.35) | <.001 | 0.03 (0.19) | 0.10 (0.34) | <.001 |
| Percent of Disabled Working Age Adults in Household | 0.02 (0.08) | 0.01 (0.06) | 0.03 (0.11) | <.001 | 0.01 (0.05) | 0.03 (0.11) | <.001 |
| Out of Pocket Healthcare Expenditures Per Household Member, $ | 1374.95 (2602.94) | 1495.98 (1872.69) | 1013.53 (4045.77) | <.001 | 1552.36 (1904.05) | 986.57 (3668.22) | <.001 |
|  |  |  |  |  |  |  |  |
| Unemployed | | | | | | | |
|  | Overall | Poverty Under Absolute Threshold | | | Poverty Under Relative Threshold | | |
|  |  | Not in Poverty | In Poverty | p-value | Not in Poverty | In Poverty | p-value |
| Weighted N | 1508477 | 742571 | 765906 |  | 641040 | 867437 |  |
|  | % or mean (SD) | % or mean (SD) | % or mean (SD) |  | % or mean (SD) | % or mean (SD) |  |
| Age, y | 38.02 (13.34) | 37.25 (13.87) | 38.76 (12.78) | 0.011 | 37.13 (13.92) | 38.68 (12.87) | 0.038 |
| Female | 38.44 | 35.25 | 41.52 | 0.001 | 35.68 | 40.47 | 0.0149 |
| Race and Ethnicity | |  |  | <.001 |  |  | <.001 |
| Non-Hispanic White | 46.49 | 54.97 | 38.26 |  | 54.06 | 40.90 |  |
| Non-Hispanic Black | 22.11 | 16.77 | 27.29 |  | 14.98 | 27.38 |  |
| Non-Hispanic American Indian and Alaskan Native | 2.16 | 1.61 | 2.69 |  | 1.30 | 2.79 |  |
| Non-Hispanic Asian | 4.71 | 6.05 | 3.40 |  | 6.76 | 3.19 |  |
| Non-Hispanic Native Hawaiian and Other Pacific Islander | 0.86 | 1.21 | 0.52 |  | 1.40 | 0.46 |  |
| Non-Hispanic Multi-Racial | 1.58 | 1.90 | 1.28 |  | 1.91 | 1.34 |  |
| Hispanic, any race | 22.09 | 17.49 | 26.55 |  | 19.58 | 23.94 |  |
| Education | |  |  | <.001 |  |  | <.001 |
| Child, education incomplete | 0.00 | 0.00 | 0.00 |  | 0.00 | 0.00 |  |
| Less than HS Diploma | 20.08 | 16.43 | 23.62 |  | 13.62 | 24.85 |  |
| HS Diploma or GED | 41.61 | 38.40 | 44.72 |  | 39.78 | 42.96 |  |
| Greater than HS Diploma | 38.31 | 45.17 | 31.66 |  | 46.60 | 32.19 |  |
| Marital Status | |  |  | <.001 |  |  | <.001 |
| Married - Civilian Spouse Present | 21.47 | 30.51 | 12.72 |  | 32.47 | 13.35 |  |
| Married - Armed Forces Spouse Present | 0.19 | 0.40 | 0.00 |  | 0.46 | 0.00 |  |
| Married - Spouse Absent | 3.66 | 1.88 | 5.39 |  | 2.44 | 4.57 |  |
| Widowed | 2.01 | 1.95 | 2.07 |  | 1.84 | 2.14 |  |
| Divorced | 10.53 | 6.15 | 14.77 |  | 5.06 | 14.57 |  |
| Separated | 3.08 | 3.58 | 2.60 |  | 1.72 | 4.09 |  |
| Never married | 59.04 | 55.54 | 62.44 |  | 56.01 | 61.29 |  |
| Personal Factor Income, $ | 771.58 (7829.58) | 1476.01 (11074.50) | 88.61 (1017.38) | <.001 | 1692.58 (11905.02) | 90.96 (974.93) | <.001 |
| No Personal Factor Income | 85.82 | 79.77 | 91.69 | <.001 | 78.90 | 90.93 | <.001 |
| SPM Resources, $ | 39808.11 (58168.62) | 69434.77 (70841.09) | 11084.11 (11262.46) | <.001 | 77246.59 (73298.65) | 12140.88 (10810.37) | <.001 |
| Household Size | 3.05 (1.66) | 3.44 (1.49) | 2.67 (1.73) | <.001 | 3.59 (1.48) | 2.65 (1.67) | <.001 |
| Number Under Age 18 in Household | 0.62 (1.08) | 0.66 (1.05) | 0.57 (1.11) | 0.02 | 0.68 (1.04) | 0.57 (1.11) | 0.006 |
| Percent Under Age 18 in Household | 0.14 (0.21) | 0.14 (0.20) | 0.13 (0.22) | 0.129 | 0.14 (0.20) | 0.13 (0.22) | 0.047 |
| Number Age 65 or Older in Household | 0.21 (0.50) | 0.27 (0.56) | 0.16 (0.42) | <.001 | 0.26 (0.56) | 0.18 (0.44) | 0.006 |
| Percent Age 65 or Older in Household | 0.07 (0.16) | 0.09 (0.17) | 0.05 (0.14) | <.001 | 0.08 (0.17) | 0.06 (0.15) | 0.047 |
| Number of Working Age Adults in Household | 2.21 (1.10) | 2.50 (1.02) | 1.93 (1.11) | <.001 | 2.65 (1.05) | 1.89 (1.04) | <.001 |
| Percent of Working Age Adults in Household | 0.80 (0.24) | 0.77 (0.23) | 0.82 (0.25) | <.001 | 0.78 (0.22) | 0.81 (0.25) | 0.003 |
| Number of Paid Laborers in Household | 0.82 (0.90) | 1.22 (0.91) | 0.44 (0.70) | <.001 | 1.35 (0.91) | 0.43 (0.65) | <.001 |
| Percent of Paid Laborers in Household | 0.24 (0.24) | 0.35 (0.22) | 0.13 (0.20) | <.001 | 0.38 (0.21) | 0.14 (0.20) | <.001 |
| Number of Disabled Working Age Adults in Household | 0.16 (0.43) | 0.14 (0.42) | 0.18 (0.44) | 0.364 | 0.12 (0.39) | 0.18 (0.46) | 0.077 |
| Percent of Disabled Working Age Adults in Household | 0.04 (0.12) | 0.04 (0.12) | 0.05 (0.12) | 0.5 | 0.03 (0.11) | 0.05 (0.13) | 0.077 |
| Out of Pocket Healthcare Expenditures Per Household Member, $ | 1142.53 (1813.00) | 1649.66 (1968.65) | 650.84 (1494.00) | <.001 | 1836.66 (2093.55) | 629.56 (1365.35) | <.001 |
|  |  |  |  |  |  |  |  |
| Other Not In Labor Force | | | | | | | |
|  | Overall | Poverty Under Absolute Threshold | | | Poverty Under Relative Threshold | | |
|  |  | Not in Poverty | In Poverty | p-value | Not in Poverty | In Poverty | p-value |
| Weighted N | 9676682 | 7138196 | 2538486 |  | 6497900 | 3178783 |  |
|  | % or mean (SD) | % or mean (SD) | % or mean (SD) |  | % or mean (SD) | % or mean (SD) |  |
| Age, y | 51.96 (13.80) | 52.86 (13.47) | 49.45 (14.39) | <.001 | 52.75 (13.56) | 50.36 (14.14) | <.001 |
| Female | 54.51 | 55.62 | 51.40 | <.001 | 56.09 | 51.28 | <.001 |
| Race and Ethnicity | |  |  | <.001 |  |  | <.001 |
| Non-Hispanic White | 64.89 | 68.69 | 54.23 |  | 68.55 | 57.41 |  |
| Non-Hispanic Black | 12.8 | 11.31 | 17.00 |  | 10.97 | 16.54 |  |
| Non-Hispanic American Indian and Alaskan Native | 0.87 | 0.71 | 1.33 |  | 0.55 | 1.54 |  |
| Non-Hispanic Asian | 7.19 | 6.61 | 8.82 |  | 6.91 | 7.76 |  |
| Non-Hispanic Native Hawaiian and Other Pacific Islander | 0.22 | 0.21 | 0.28 |  | 0.31 | 0.04 |  |
| Non-Hispanic Multi-Racial | 1.33 | 1.05 | 2.11 |  | 1.15 | 1.68 |  |
| Hispanic, any race | 12.7 | 11.44 | 16.24 |  | 11.56 | 15.02 |  |
| Education | |  |  | <.001 |  |  | <.001 |
| Child, education incomplete | 0.00 | 0.00 | 0.00 |  | 0.00 | 0.00 |  |
| Less than HS Diploma | 10.62 | 8.97 | 15.27 |  | 8.41 | 15.14 |  |
| HS Diploma or GED | 31.67 | 30.88 | 33.90 |  | 29.85 | 35.40 |  |
| Greater than HS Diploma | 57.71 | 60.16 | 50.83 |  | 61.74 | 49.47 |  |
| Marital Status | |  |  | <.001 |  |  | <.001 |
| Married - Civilian Spouse Present | 58.33 | 64.84 | 40.01 |  | 66.59 | 41.44 |  |
| Married - Armed Forces Spouse Present | 0.21 | 0.22 | 0.18 |  | 0.24 | 0.14 |  |
| Married - Spouse Absent | 1.79 | 1.53 | 2.52 |  | 1.48 | 2.43 |  |
| Widowed | 6.49 | 5.83 | 8.34 |  | 5.46 | 8.59 |  |
| Divorced | 10.28 | 8.88 | 14.23 |  | 8.05 | 14.84 |  |
| Separated | 2.05 | 1.41 | 3.84 |  | 1.32 | 3.53 |  |
| Never married | 20.85 | 17.28 | 30.87 |  | 16.85 | 29.01 |  |
| Personal Factor Income, $ | 7380.44 (47128.25) | 9037.46 (49565.00) | 2720.93 (39121.50) | <.001 | 9799.64 (51855.89) | 2435.25 (35059.10) | <.001 |
| No Personal Factor Income | 45.81 | 39.53 | 63.52 | <.001 | 37.43 | 62.97 | <.001 |
| SPM Resources, $ | 56589.23 (71299.05) | 74054.30 (74958.86) | 7477.64 (17587.45) | <.001 | 79614.85 (76355.37) | 9521.47 (16089.95) | <.001 |
| Household Size | 2.49 (1.38) | 2.61 (1.36) | 2.18 (1.39) | <.001 | 2.69 (1.39) | 2.10 (1.28) | <.001 |
| Number Under Age 18 in Household | 0.31 (0.80) | 0.31 (0.79) | 0.32 (0.83) | 0.467 | 0.32 (0.80) | 0.29 (0.79) | 0.03 |
| Percent Under Age 18 in Household | 0.07 (0.16) | 0.07 (0.15) | 0.07 (0.17) | 0.024 | 0.07 (0.15) | 0.07 (0.16) | 0.656 |
| Number Age 65 or Older in Household | 0.23 (0.45) | 0.25 (0.46) | 0.16 (0.40) | <.001 | 0.25 (0.47) | 0.17 (0.41) | <.001 |
| Percent Age 65 or Older in Household | 0.09 (0.19) | 0.10 (0.20) | 0.06 (0.16) | <.001 | 0.10 (0.19) | 0.08 (0.18) | <.001 |
| Number of Working Age Adults in Household | 1.96 (0.96) | 2.05 (0.98) | 1.70 (0.86) | <.001 | 2.11 (1.00) | 1.64 (0.79) | <.001 |
| Percent of Working Age Adults in Household | 0.84 (0.22) | 0.83 (0.23) | 0.86 (0.22) | <.001 | 0.83 (0.22) | 0.86 (0.23) | <.001 |
| Number of Paid Laborers in Household | 0.65 (0.85) | 0.79 (0.89) | 0.26 (0.59) | <.001 | 0.86 (0.91) | 0.23 (0.52) | <.001 |
| Percent of Paid Laborers in Household | 0.21 (0.25) | 0.26 (0.25) | 0.08 (0.18) | <.001 | 0.28 (0.25) | 0.08 (0.17) | <.001 |
| Number of Disabled Working Age Adults in Household | 0.06 (0.26) | 0.06 (0.25) | 0.07 (0.28) | 0.001 | 0.05 (0.24) | 0.08 (0.29) | <.001 |
| Percent of Disabled Working Age Adults in Household | 0.02 (0.09) | 0.02 (0.09) | 0.02 (0.10) | 0.001 | 0.02 (0.08) | 0.03 (0.11) | <.001 |
| Out of Pocket Healthcare Expenditures Per Household Member, $ | 2248.62 (2912.57) | 2540.15 (2916.96) | 1428.84 (2739.71) | <.001 | 2604.64 (2940.70) | 1520.86 (2713.39) | <.001 |
|  |  |  |  |  |  |  |  |
| Paid Laborer | | | | | | | |
|  | Overall | Poverty Under Absolute Threshold | | | Poverty Under Relative Threshold | | |
|  |  | Not in Poverty | In Poverty | p-value | Not in Poverty | In Poverty | p-value |
| Weighted N | 147908235 | 137483761 | 10424475 |  | 132535189 | 15373046 |  |
|  | % or mean (SD) | % or mean (SD) | % or mean (SD) |  | % or mean (SD) | % or mean (SD) |  |
| Age, y | 40.89 (12.83) | 41.12 (12.77) | 37.87 (13.27) | <.001 | 41.22 (12.74) | 38.05 (13.24) | <.001 |
| Female | 48.00 | 47.84 | 50.18 | <.001 | 47.65 | 51.02 | <.001 |
| Race and Ethnicity | |  |  | <.001 |  |  | <.001 |
| Non-Hispanic White | 61.98 | 63.70 | 39.28 |  | 63.82 | 46.14 |  |
| Non-Hispanic Black | 11.41 | 10.98 | 17.05 |  | 10.72 | 17.32 |  |
| Non-Hispanic American Indian and Alaskan Native | 0.69 | 0.68 | 0.91 |  | 0.63 | 1.22 |  |
| Non-Hispanic Asian | 6.42 | 6.43 | 6.37 |  | 6.63 | 4.66 |  |
| Non-Hispanic Native Hawaiian and Other Pacific Islander | 0.33 | 0.31 | 0.52 |  | 0.32 | 0.41 |  |
| Non-Hispanic Multi-Racial | 1.44 | 1.42 | 1.75 |  | 1.42 | 1.60 |  |
| Hispanic, any race | 17.73 | 16.49 | 34.11 |  | 16.46 | 28.65 |  |
| Education | |  |  | <.001 |  |  | <.001 |
| Child, education incomplete | 0.00 | 0.00 | 0.00 |  | 0.00 | 0.00 |  |
| Less than HS Diploma | 7.29 | 6.22 | 21.38 |  | 5.92 | 19.07 |  |
| HS Diploma or GED | 25.84 | 25.18 | 34.61 |  | 24.74 | 35.33 |  |
| Greater than HS Diploma | 66.87 | 68.60 | 44.00 |  | 69.34 | 45.60 |  |
| Marital Status | |  |  | <.001 |  |  | <.001 |
| Married - Civilian Spouse Present | 53.12 | 54.77 | 31.39 |  | 55.61 | 31.66 |  |
| Married - Armed Forces Spouse Present | 0.32 | 0.33 | 0.13 |  | 0.34 | 0.11 |  |
| Married - Spouse Absent | 1.55 | 1.47 | 2.71 |  | 1.42 | 2.68 |  |
| Widowed | 1.25 | 1.20 | 1.84 |  | 1.16 | 2.01 |  |
| Divorced | 9.67 | 9.64 | 10.04 |  | 9.43 | 11.74 |  |
| Separated | 2.02 | 1.85 | 4.26 |  | 1.73 | 4.56 |  |
| Never married | 32.07 | 30.74 | 49.63 |  | 30.31 | 47.24 |  |
| Personal Factor Income, $ | 58689.02 (79649.57) | 61918.69 (81198.38) | 16094.34 (33269.28) | <.001 | 63513.23 (82248.39) | 17098.20 (28042.99) | <.001 |
| No Personal Factor Income | -- | -- | -- | -- | -- | -- | -- |
| SPM Resources, $ | 83419.14 (78259.64) | 88468.25 (78814.15) | 16828.78 (14332.30) | <.001 | 91004.39 (79148.73) | 18024.66 (12065.81) | <.001 |
| Household Size | 3.01 (1.56) | 3.02 (1.54) | 2.89 (1.79) | <.001 | 3.04 (1.54) | 2.74 (1.70) | <.001 |
| Number Under Age 18 in Household | 0.77 (1.11) | 0.76 (1.10) | 0.86 (1.25) | <.001 | 0.75 (1.09) | 0.89 (1.27) | <.001 |
| Percent Under Age 18 in Household | 0.18 (0.23) | 0.18 (0.23) | 0.20 (0.25) | <.001 | 0.17 (0.23) | 0.21 (0.26) | <.001 |
| Number Age 65 or Older in Household | 0.10 (0.34) | 0.10 (0.34) | 0.09 (0.33) | <.001 | 0.10 (0.35) | 0.07 (0.30) | <.001 |
| Percent Age 65 or Older in Household | 0.03 (0.11) | 0.03 (0.11) | 0.03 (0.10) | <.001 | 0.03 (0.11) | 0.03 (0.10) | <.001 |
| Number of Working Age Adults in Household | 2.15 (0.96) | 2.16 (0.96) | 1.94 (1.01) | <.001 | 2.19 (0.97) | 1.78 (0.86) | <.001 |
| Percent of Working Age Adults in Household | 0.79 (0.24) | 0.79 (0.24) | 0.78 (0.26) | 0.954 | 0.79 (0.24) | 0.77 (0.27) | <.001 |
| Number of Paid Laborers in Household | 1.95 (0.88) | 1.98 (0.88) | 1.47 (0.71) | <.001 | 2.01 (0.89) | 1.40 (0.63) | <.001 |
| Percent of Paid Laborers in Household | 0.74 (0.27) | 0.74 (0.26) | 0.65 (0.31) | <.001 | 0.75 (0.26) | 0.66 (0.31) | <.001 |
| Number of Disabled Working Age Adults in Household | 0.04 (0.22) | 0.04 (0.21) | 0.09 (0.31) | <.001 | 0.04 (0.21) | 0.08 (0.29) | <.001 |
| Percent of Disabled Working Age Adults in Household | 0.01 (0.07) | 0.01 (0.07) | 0.03 (0.10) | <.001 | 0.01 (0.07) | 0.03 (0.10) | <.001 |
| Out of Pocket Healthcare Expenditures Per Household Member, $ | 2016.83 (2645.81) | 2039.60 (2329.95) | 1716.45 (5257.01) | <.001 | 2061.86 (2329.90) | 1628.54 (4515.09) | <.001 |
| All numbers weighted to be nationally-representative | | | | | | | |

| Table S2: Specific Analyses for Children, 2019 ASEC Data | | | | | | | |
| --- | --- | --- | --- | --- | --- | --- | --- |
|  | Overall | Poverty Under Absolute Threshold | | | Poverty Under Relative Threshold | | |
|  |  | Not in Poverty | In Poverty | p-value | Not in Poverty | In Poverty | p-value |
| Weighted N | 71522953 | 61924007 | 9598947 |  | 57721153 | 13801801 |  |
|  | mean (SD) | mean (SD) | mean (SD) |  | mean (SD) | mean (SD) |  |
| Value of Child Benefits, per child | 887.63 (1355.42) | 797.05 (1289.38) | 1471.95 (1603.14) | <.001 | 684.18 (1199.30) | 1738.47 (1615.09) | <.001 |
| Childcare costs, per child | 750.30 (2638.78) | 805.35 (2409.57) | 395.17 (3779.50) | <.001 | 841.26 (2479.01) | 369.87 (3194.44) | <.001 |
| All numbers weighted to be nationally-representative P-values are from t-tests for continuous variables and chi-squared tests for categorical variables | | | | | | | |

| Table S3: Specific Analyses for Older Adults, 2019 ASEC Data | | | | | | | |
| --- | --- | --- | --- | --- | --- | --- | --- |
|  | Overall | Poverty Under Absolute Threshold | | | Poverty Under Relative Threshold | | |
|  |  | Not in Poverty | In Poverty | p-value | Not in Poverty | In Poverty | p-value |
| Weighted N | 53031626 | 45869024 | 7162602 |  | 40478135 | 12553491 |  |
|  | % (weighted N) or mean (SD) | % (weighted N) or mean (SD) | % (weighted N) or mean (SD) |  | % (weighted N) or mean (SD) | % (weighted N) or mean (SD) |  |
| In Paid Labor Force | 23.15 (12276317.39) | 25.50 (11698339.36) | 8.07 (577978.03) | <.001 | 27.89 (11288974.11) | 7.87 (987343.28) | <.001 |
| Any Annuity Income | 6.56 (3480570.94) | 7.32 (3355609.77) | 1.74 (124961.17) | <.001 | 8.01 (3240964.51) | 1.91 (239606.43) | <.001 |
| Amount of Annuity Income, if any, $ | 11790.80 (18042.04) | 12033.14 (18277.76) | 5283.28 (7194.20) | <.001 | 12301.77 (18519.79) | 4879.26 (6165.29) | <.001 |
| Any Retirement Distribution Income | 17.48 (9267579.01) | 19.61 (8997029.82) | 3.78 (270549.19) | <.001 | 21.59 (8739476.48) | 4.21 (528102.53) | <.001 |
| Amount of Retirement Distribution Income, if any, $ | 16269.41 (44454.41) | 16646.50 (45056.19) | 3729.19 (4831.29) | <.001 | 17052.18 (45649.08) | 3315.45 (4159.22) | <.001 |
| Any Pension Income | 26.61 (14111867.02) | 29.64 (13594401.94) | 7.22 (517465.08) | <.001 | 32.13 (13006106.85) | 8.81 (1105760.17) | <.001 |
| Amount of Pension Income, if any, $ | 22232.81 (29879.89) | 22870.50 (30230.16) | 5480.09 (6963.05) | <.001 | 23641.01 (30656.35) | 5669.43 (6511.54) | <.001 |
| Any Retirement Interest Income | 31.97 (16951875.05) | 35.57 (16317692.77) | 8.85 (634182.28) | <.001 | 39.05 (15806757.94) | 9.12 (1145117.11) | <.001 |
| Amount of Retirement Interest Income, if any, $ | 5108.00 (14522.18) | 5273.58 (14772.33) | 847.43 (1876.49) | <.001 | 5422.59 (14982.62) | 765.55 (1785.08) | <.001 |
| Any Social Security Income | 80.79 (42843799.85) | 83.04 (38090473.35) | 66.36 (4753326.50) | <.001 | 81.91 (33156798.10) | 77.17 (9687001.75) | <.001 |
| Amount of Social Security Income, if any, $ | 16060.54 (7896.86) | 16919.57 (7730.52) | 9176.77 (5467.98) | <.001 | 17461.15 (7938.70) | 11266.51 (5513.62) | <.001 |
| Any Supplemental Security Income | 2.75 (1456647.52) | 2.08 (953936.39) | 7.02 (502711.13) | <.001 | 1.70 (689311.50) | 6.11 (767336.02) | <.001 |
| Amount of Supplemental Security Income, if any, $ | 6634.24 (4909.36) | 7247.70 (5179.00) | 5462.50 (4101.04) | <.001 | 7824.19 (5396.46) | 5555.57 (4136.00) | <.001 |
| Any Survival Benefit Income | 4.10 (2174859.38) | 4.42 (2027973.85) | 2.05 (146885.53) | <.001 | 4.59 (1858783.58) | 2.52 (316075.80) | <.001 |
| Amount of Survival Benefit Income, if any, $ | 15594.21 (19647.31) | 16211.07 (19659.20) | 7077.64 (17374.61) | <.001 | 17251.19 (20159.59) | 5849.81 (12451.43) | <.001 |
| Any Veteran's Payment Income | 3.97 (2103179.27) | 4.33 (1986479.26) | 1.63 (116700.01) | <.001 | 4.62 (1869857.15) | 1.86 (233322.12) | <.001 |
| Amount of Veteran's Payment Income, if any, $ | 15364.64 (15340.28) | 15966.11 (15543.47) | 5126.36 (4189.45) | <.001 | 16602.90 (15740.13) | 5441.14 (4999.02) | <.001 |
| Any Income from Defined Benefit Retirement Plan | 35.26 (18701455.42) | 38.95 (17864752.95) | 11.68 (836702.47) | <.001 | 41.81 (16924895.76) | 14.15 (1776559.66) | <.001 |
| Any Income from Defined Contribution Retirement Plan | 31.97 (16951875.05) | 35.57 (16317692.77) | 8.85 (634182.28) | <.001 | 39.05 (15806757.94) | 9.12 (1145117.11) | <.001 |
| Retirement Income Category | | |  | <.001 |  |  | <.001 |
| No Retirement Income | 10.96 (5813244.62) | 8.19 (3756032.96) | 28.72 (2057211.66) |  | 8.49 (3438288.20) | 18.92 (2374956.42) |  |
| Social Security Income Alone | 36.82 (19527117.34) | 34.34 (15752325.20) | 52.70 (3774792.14) |  | 29.67 (12010769.76) | 59.87 (7516347.58) |  |
| Defined Benefit Income Alone | 2.12 (1123655.16) | 2.01 (921491.90) | 2.82 (202163.26) |  | 2.10 (849736.04) | 2.18 (273919.12) |  |
| Defined Contribution Income Alone | 4.47 (2372642.61) | 4.88 (2240094.67) | 1.85 (132547.94) |  | 5.39 (2182431.07) | 1.52 (190211.54) |  |
| Social Security and Defined Benefit Income | 18.13 (9615733.84) | 19.89 (9121481.16) | 6.90 (494252.68) |  | 20.68 (8372583.55) | 9.90 (1243150.29) |  |
| Social Security and Defined Contribution Income | 12.48 (6617166.02) | 13.64 (6255818.21) | 5.04 (361347.81) |  | 14.63 (5921750.70) | 5.54 (695415.32) |  |
| Defined Benefit and Defined Contribution Income | 1.66 (878283.77) | 1.88 (860931.11) | 0.24 (17352.66) |  | 2.10 (850882.08) | 0.22 (27401.69) |  |
| Social Security, Defined Benefit, and Defined Contribution Income | 13.36 (7083782.65) | 15.18 (6960848.78) | 1.72 (122933.87) |  | 16.93 (6851694.09) | 1.85 (232088.56) |  |
| Total Amount of Retirement Income, $ | 25390.09 (34276.96) | 28258.23 (35889.40) | 7022.63 (7770.45) | <.001 | 30242.37 (37696.92) | 9744.13 (7781.89) | <.001 |
| Individual Social Security Income Above Absolute Poverty Threshold | 52.93 (28070220.17) | 58.70 (26925272.89) | 15.99 (1144947.28) | <.001 | 59.20 (23962188.90) | 32.72 (4108031.27) | <.001 |
| Individual Social Security Income Above Relative Poverty Threshold | 36.37 (19288839.51) | 41.02 (18816242.94) | 6.60 (472596.57) | <.001 | 43.51 (17611098.08) | 13.36 (1677741.43) | <.001 |
| All numbers weighted to be nationally-representative  P-values are from t-tests for continuous variables and chi-squared tests for categorical variables | | | | | | | |

| Table S4: Specific Analyses for Disabled Working-Age Adults, 2019 ASEC Data | | | | | | | |
| --- | --- | --- | --- | --- | --- | --- | --- |
|  | Overall | Poverty Under Absolute Threshold | | | Poverty Under Relative Threshold | | |
|  |  | Not in Poverty | In Poverty | p-value | Not in Poverty | In Poverty | p-value |
| Weighted N | 11673462 | 7874685 | 3798777 |  | 6061502 | 5611960 |  |
|  | % (weighted N) or mean (SD) | % (weighted N) or mean (SD) | % (weighted N) or mean (SD) |  | % (weighted N) or mean (SD) | % (weighted N) or mean (SD) |  |
| Any Disability Income | 67.81 (7916343.31) | 74.58 (5872697.81) | 53.80 (2043645.50) | <.001 | 72.23 (4378284.27) | 63.04 (3538059.04) | <.001 |
| Total Amount of Disability Income, if any, $ | 14475.26 (10936.81) | 16280.84 (11916.97) | 9292.36 (4373.06) | <.001 | 17646.65 (13313.24) | 10559.53 (4553.98) | <.001 |
| Any Social Security Disability Insurance Income | 39.92 (4659811.30) | 46.02 (3623929.83) | 27.27 (1035881.47) | <.001 | 43.86 (2658660.30) | 35.66 (2001151.00) | <.001 |
| Amount of Social Security Disability Insurance Income, if any, $ | 13579.17 (6690.91) | 14759.77 (6696.40) | 9448.97 (4753.11) | <.001 | 15488.38 (7227.86) | 11042.66 (4855.37) | <.001 |
| Any Supplemental Security Income | 28.77 (3358850.11) | 29.38 (2313675.92) | 27.51 (1045174.19) | 0.114 | 27.56 (1670836.17) | 30.08 (1688013.94) | 0.002 |
| Amount of Supplemental Security Income, if any, $ | 8754.63 (4961.78) | 9168.77 (5314.97) | 7831.31 (3911.68) | <.001 | 9616.77 (5594.10) | 7894.53 (4060.28) | <.001 |
| Any Veteran's Disability Income | 2.23 (259932.27) | 2.97 (233880.36) | 0.69 (26051.91) | <.001 | 3.60 (217916.97) | 0.75 (42015.30) | <.001 |
| Amount of Veteran's Disability Income, if any, $ | 24464.62 (16874.54) | 26410.21 (16492.01) | 5864.19 (4974.82) | <.001 | 27853.27 (15902.05) | 4865.26 (4324.46) | <.001 |
| Any Worker's Compensation Income | 1.45 (169340.42) | 1.86 (146819.49) | 0.59 (22520.93) | <.001 | 2.42 (146821.11) | 0.40 (22519.31) | <.001 |
| Amount of Worker's Compensation Income, if any, $ | 16796.67 (11674.47) | 17961.85 (11758.27) | 9200.59 (7550.78) | <.001 | 17982.86 (11754.78) | 9063.01 (7427.66) | <.001 |
| Self-Report of Problem with Dressing | 13.23 (1543935.67) | 13.80 (1086954.46) | 12.03 (456981.21) | 0.003 | 13.46 (815757.61) | 12.98 (728178.06) | 0.511 |
| Self-Report of Problem with Hearing | 6.14 (716785.08) | 6.51 (512674.24) | 5.37 (204110.84) | 0.001 | 5.77 (349539.26) | 6.54 (367245.82) | 0.025 |
| Self-Report of Problem with Seeing | 7.44 (868974.34) | 7.17 (564766.58) | 8.01 (304207.76) | 0.025 | 6.27 (380179.01) | 8.71 (488795.33) | <.001 |
| Self-Report of Problem with Doing Errands | 27.21 (3176237.84) | 28.48 (2242506.49) | 24.58 (933731.35) | <.001 | 28.74 (1742243.12) | 25.55 (1433994.72) | 0.002 |
| Self-Report of Problem with Walking or Climbing Stairs | 36.86 (4302616.33) | 37.45 (2949429.10) | 35.62 (1353187.23) | 0.033 | 35.62 (2159300.81) | 38.19 (2143315.52) | 0.001 |
| Self-Report of Problem with Concentration | 26.59 (3104149.28) | 26.96 (2122865.96) | 25.83 (981283.32) | 0.204 | 26.19 (1587788.80) | 27.02 (1516360.48) | 0.357 |
| All numbers weighted to be nationally-representative  P-values are from t-tests for continuous variables and chi-squared tests for categorical variables | | | | | | | |

| Table S5: Specific Analyses for Students, 2019 ASEC Data | | | | | | | |
| --- | --- | --- | --- | --- | --- | --- | --- |
|  | Overall | Poverty Under Absolute Threshold | | | Poverty Under Relative Threshold | | |
|  |  | Not in Poverty | In Poverty | p-value | Not in Poverty | In Poverty | p-value |
| Weighted N | 8688644 | 6073795 | 2614850 |  | 5882771 | 2805873 |  |
|  | % (weighted N) or mean (SD) | % (weighted N) or mean (SD) | % (weighted N) or mean (SD) |  | % (weighted N) or mean (SD) | % (weighted N) or mean (SD) |  |
| Any Income for Education | 18.22 (1582757.45) | 20.41 (1239580.98) | 13.12 (343176.47) | <.001 | 20.37 (1198221.15) | 13.70 (384536.30) | <.001 |
| Amount of Income for Education, if any, $ | 10273.53 (13583.49) | 11918.40 (14783.99) | 4332.13 (4060.96) | <.001 | 12021.04 (14952.85) | 4828.28 (4862.90) | <.001 |
| Any Veteran's Income for Education | 0.54 (46939.57) | 0.67 (40812.55) | 0.23 (6127.02) | 0.001 | 0.68 (40057.91) | 0.25 (6881.66) | <.001 |
| Amount of Veteran's Income for Education, if any, $ | 21338.36 (11618.83) | 23175.77 (11340.02) | 9099.19 (2321.09) | <.001 | 23431.52 (11290.76) | 9154.11 (2195.65) | <.001 |
| Any Income from Financial Assistance | 6.34 (551263.48) | 5.53 (336160.71) | 8.23 (215102.77) | <.001 | 4.79 (281671.06) | 9.61 (269592.42) | <.001 |
| Amount of Financial Assistance Income, if any, $ | 15720.81 (15887.50) | 22261.58 (17032.92) | 5498.95 (4706.54) | <.001 | 24082.94 (18016.80) | 6984.02 (5254.46) | <.001 |
| All numbers weighted to be nationally-representative  P-values are from t-tests for continuous variables and chi-squared tests for categorical variables | | | | | | | |

| Table S6: Specific Analyses for Caregivers, 2019 ASEC Data | | | | | | | |
| --- | --- | --- | --- | --- | --- | --- | --- |
|  | Overall | Poverty Under Absolute Threshold | | | Poverty Under Relative Threshold | | |
|  |  | Not in Poverty | In Poverty | p-value | Not in Poverty | In Poverty | p-value |
| Weighted N | 12756769 | 9556609 | 3200160 |  | 8756843 | 3999925 |  |
|  | % (weighted N) or mean (SD) | % (weighted N) or mean (SD) | % (weighted N) or mean (SD) |  | % (weighted N) or mean (SD) | % (weighted N) or mean (SD) |  |
| Any Income from Financial Assistance | 1.01 (129322.66) | 0.67 (64114.84) | 2.04 (65207.82) | <.001 | 0.68 (59378.57) | 1.75 (69944.09) | <.001 |
| Amount of Financial Assistance Income, if any, $ | 14916.17 (27447.93) | 25942.59 (35605.51) | 4074.57 (3254.03) | <.001 | 27770.86 (36381.04) | 4003.27 (3160.83) | <.001 |
| Any Child Support Income | 2.59 (330823.12) | 2.18 (208550.79) | 3.82 (122272.33) | <.001 | 1.87 (163471.74) | 4.18 (167351.38) | <.001 |
| Amount of Child Support Income, if any, $ | 6100.80 (8661.32) | 7468.29 (10282.36) | 3768.38 (3744.88) | <.001 | 7884.77 (11144.74) | 4358.18 (4565.00) | <.001 |
| Any TANF or Other Cash Welfare Income | 1.93 (245996.05) | 1.13 (108248.43) | 4.30 (137747.62) | <.001 | 1.14 (99971.77) | 3.65 (146024.28) | <.001 |
| Amount of TANF or Other Cash Welfare Income, if any, $ | 4052.71 (3360.84) | 3961.69 (3827.36) | 4124.25 (2941.49) | 0.593 | 4141.89 (4025.74) | 3991.66 (2815.43) | 0.645 |
| Type of Cash Welfare Income | | |  | <.001 |  |  | <.001 |
| None | 98.07 (12510772.63) | 98.87 (9448360.48) | 95.70 (3062412.15) |  | 98.86 (8656871.51) | 96.35 (3853901.12) |  |
| TANF | 1.32 (168121.56) | 0.62 (58959.21) | 3.41 (109162.35) |  | 0.56 (49249.03) | 2.97 (118872.53) |  |
| Other | 0.61 (77874.49) | 0.52 (49289.22) | 0.89 (28585.27) |  | 0.58 (50722.74) | 0.68 (27151.75) |  |
| All numbers weighted to be nationally-representative  P-values are from t-tests for continuous variables and chi-squared tests for categorical variables | | | | | | | |

| Table S7: Specific Analyses for Long-Term Unemployed Working-Age Adults, 2019 ASEC Data | | | | | | | |
| --- | --- | --- | --- | --- | --- | --- | --- |
|  | Overall | Poverty Under Absolute Threshold | | | Poverty Under Relative Threshold | | |
|  |  | Not in Poverty | In Poverty | p-value | Not in Poverty | In Poverty | p-value |
| Weighted N | 1508477 | 742571 | 765906 |  | 641040 | 867437 |  |
|  | % (weighted N) or mean (SD) | % (weighted N) or mean (SD) | % (weighted N) or mean (SD) |  | % (weighted N) or mean (SD) | % (weighted N) or mean (SD) |  |
| Any Unemployment Insurance Income | 6.75 (101800.57) | 8.37 (62126.78) | 5.18 (39673.79) | 0.002 | 8.21 (52655.42) | 5.67 (49145.15) | 0.02 |
| Amount of Unemployment Insurance Income, if any, $ | 5620.11 (4277.87) | 6548.70 (4481.68) | 4166.00 (3477.09) | <.001 | 6612.73 (4587.99) | 4556.60 (3635.15) | 0.003 |
| Self-Report of a Disability that Limits Work | 4.84 (73066.01) | 7.05 (52362.61) | 2.70 (20703.40) | <.001 | 5.43 (34826.66) | 4.41 (38239.35) | 0.303 |
| Any Disability Income | 3.55 (53560.14) | 6.67 (49556.37) | 0.52 (4003.77) | <.001 | 6.25 (40083.59) | 1.55 (13476.55) | <.001 |
| Total Amount of Disability Income, if any, $ | 11327.33 (8764.65) | 11984.97 (9300.08) | 7429.36 (1540.69) | <.001 | 12624.86 (10079.84) | 8853.55 (4535.24) | 0.025 |
| All numbers weighted to be nationally-representative  P-values are from t-tests for continuous variables and chi-squared tests for categorical variables | | | | | | | |

| Table S8: Specific Analyses for Working-Age Adults not in the Paid Labor Force for Other Reasons, 2019 ASEC Data | | | | | | | |
| --- | --- | --- | --- | --- | --- | --- | --- |
|  | Overall | Poverty Under Absolute Threshold | | | Poverty Under Relative Threshold | | |
|  |  | Not in Poverty | In Poverty | p-value | Not in Poverty | In Poverty | p-value |
| Weighted N | 9676682 | 7138196 | 2538486 |  | 6497900 | 3178783 |  |
|  | % (weighted N) or mean (SD) | % (weighted N) or mean (SD) | % (weighted N) or mean (SD) |  | % (weighted N) or mean (SD) | % (weighted N) or mean (SD) |  |
| Reason For Not Being in Paid Labor Force in Past Year | | | | <.001 |  |  | <.001 |
| Not Asked | 11.50 (1113072.22) | 12.40 (885108.41) | 8.98 (227963.81) |  | 13.02 (845881.24) | 8.41 (267190.98) |  |
| Retired | 75.33 (7289049.72) | 78.08 (5573655.52) | 67.58 (1715394.20) |  | 77.49 (5035204.12) | 70.90 (2253845.60) |  |
| Other | 13.17 (1274560.32) | 9.52 (679431.96) | 23.44 (595128.36) |  | 9.49 (616814.32) | 20.69 (657746.00) |  |
| Any Early Retirement Income | 1.91 (185235.57) | 2.14 (153068.43) | 1.27 (32167.14) | 0.007 | 2.30 (149574.93) | 1.12 (35660.64) | <.001 |
| Self-Report of a Disability that Limits Work | 10.84 (1049065.55) | 11.22 (800892.92) | 9.78 (248172.63) | 0.024 | 10.55 (685370.97) | 11.44 (363694.58) | 0.113 |
| Any Disability Income | 8.77 (848984.34) | 10.15 (724453.76) | 4.91 (124530.58) | <.001 | 9.69 (629748.91) | 6.90 (219235.43) | <.001 |
| Total Amount of Disability Income, if any, $ | 18415.90 (15130.73) | 20212.16 (15612.68) | 8184.60 (4644.75) | <.001 | 21411.93 (16373.22) | 10000.61 (4798.73) | <.001 |
| Any Rental Income | 5.24 (507211.62) | 6.25 (445956.94) | 2.41 (61254.68) | <.001 | 6.50 (422425.17) | 2.67 (84786.45) | <.001 |
| Amount of Rental Income, if any, $ | 18396.57 (57687.88) | 20573.62 (61190.03) | 2546.85 (3611.77) | <.001 | 21235.76 (62785.15) | 4251.08 (5480.11) | <.001 |
| Any Capital Income | 49.44 (4784374.28) | 56.13 (4006526.46) | 30.64 (777847.82) | <.001 | 57.98 (3767356.77) | 31.99 (1017017.51) | <.001 |
| Amount of Capital Income, if any, $ | 7910.90 (56237.31) | 7969.04 (52440.65) | 7616.05 (72521.37) | 0.893 | 8384.64 (54011.16) | 6173.98 (63729.65) | 0.299 |
| Any Income from Financial Assistance | 1.03 (99764.36) | 0.76 (54320.94) | 1.79 (45443.42) | <.001 | 0.79 (51308.97) | 1.52 (48455.39) | <.001 |
| Amount of Financial Assistance Income, if any, $ | 7592.17 (11813.19) | 11800.44 (14506.08) | 2561.80 (2893.19) | <.001 | 12000.05 (14901.88) | 2924.70 (3136.43) | <.001 |
| All numbers weighted to be nationally-representative  P-values are from t-tests for continuous variables and chi-squared tests for categorical variables | | | | | | | |

| Table S9: Specific Analyses for Paid Laborers, 2019 ASEC Data | | | | | | | |
| --- | --- | --- | --- | --- | --- | --- | --- |
|  | Overall | Poverty Under Absolute Threshold | | | Poverty Under Relative Threshold | | |
|  | Overall | Not in Poverty | In Poverty | P-value | Not in Poverty | In Poverty | P-value |
| Weighted N | 147908235 | 137483761 | 10424475 |  | 132535189 | 15373046 |  |
|  | % (weighted N) or mean (SD) | % (weighted N) or mean (SD) | % (weighted N) or mean (SD) |  | % (weighted N) or mean (SD) | % (weighted N) or mean (SD) |  |
| Individual Personal Income Above Absolute Poverty Threshold | 87.96 (130095991.66) | 90.82 (124861810.54) | 50.21 (5234181.12) | <.001 | 91.39 (121121874.33) | 58.38 (8974117.33) | <.001 |
| Individual Personal Income Above Relative Poverty Threshold | 83.86 (124034129.50) | 87.51 (120316214.17) | 35.67 (3717915.33) | <.001 | 88.42 (117181423.39) | 44.58 (6852706.11) | <.001 |
| Number of Weeks in Paid Labor Force in the Past Year | 48.25 (9.91) | 48.69 (9.26) | 42.41 (15.01) | <.001 | 48.80 (9.11) | 43.45 (14.29) | <.001 |
| Looking for Work or on Layoff at any Time in Past Year | 6.14 (9085947.25) | 5.43 (7469511.19) | 15.51 (1616436.06) | <.001 | 5.18 (6869471.85) | 14.42 (2216475.40) | <.001 |
| Number of Weeks Looking for Work or on Layoff at any Time in Past Year | 1.04 (5.13) | 0.86 (4.60) | 3.33 (9.45) | <.001 | 0.83 (4.51) | 2.86 (8.64) | <.001 |
| Self-Report of Leaving Job Owing to Illness | 2.12 (3136568.23) | 1.90 (2610709.46) | 5.04 (525858.77) | <.001 | 1.77 (2341031.72) | 5.17 (795536.51) | <.001 |
| Self-Report of Leaving Job Owing to Caregiving | 2.63 (3887231.35) | 2.41 (3312955.39) | 5.51 (574275.96) | <.001 | 2.32 (3071977.21) | 5.30 (815254.14) | <.001 |
| Any Unemployment Insurance Income | 1.73 (2563541.14) | 1.76 (2415123.54) | 1.42 (148417.60) | 0.002 | 1.71 (2262607.88) | 1.96 (300933.26) | <.001 |
| Amount of Unemployment Insurance Income, if any, $ | 4404.94 (4368.82) | 4458.62 (4441.76) | 3531.49 (2796.54) | <.001 | 4575.61 (4525.44) | 3121.79 (2597.70) | <.001 |
| Self-Report of a Disability that Limits Work | 3.14 (4651582.77) | 3.01 (4143005.81) | 4.88 (508576.96) | <.001 | 2.87 (3809418.81) | 5.48 (842163.96) | <.001 |
| Any Disability Income | 2.20 (3253650.54) | 2.25 (3093431.66) | 1.54 (160218.88) | <.001 | 2.21 (2928323.32) | 2.12 (325327.22) | 0.129 |
| Total Amount of Disability Income, if any, $ | 11121.87 (11906.09) | 11413.69 (12066.73) | 6117.02 (7014.96) | <.001 | 11639.14 (12338.74) | 6911.03 (6038.25) | <.001 |
| Any Worker's Compensation Income | 0.39 (576003.00) | 0.38 (528051.97) | 0.46 (47951.03) | 0.258 | 0.38 (504466.93) | 0.47 (71536.07) | 0.125 |
| Amount of Worker's Compensation Income, if any, $ | 6295.29 (9099.43) | 6688.17 (9352.01) | 1968.81 (3325.75) | <.001 | 6887.18 (9505.29) | 2121.32 (3108.00) | <.001 |
| Number of Weeks Looking for Work or on Layoff at any Time in Past Year, Among Those Who Report Looking for Work or Being on Layoff at any Time in Past Year | 16.89 (12.70) | 15.90 (12.26) | 21.47 (13.67) | <.001 | 15.95 (12.30) | 19.82 (13.46) | <.001 |
| Any Unemployment Insurance Income, Among Those Who Report Looking for Work or Being on Layoff at any Time in Past Year | 16.13 (1465608.43) | 18.32 (1368228.95) | 6.02 (97379.48) | <.001 | 18.76 (1288456.00) | 7.99 (177152.43) | <.001 |
| Amount of Unemployment Insurance Income, if any, $, Among Those Who Report Looking for Work or Being on Layoff at any Time in Past Year | 5208.94 (4610.46) | 5284.42 (4692.46) | 4148.41 (3058.74) | <.001 | 5416.89 (4764.60) | 3696.50 (2856.50) | <.001 |
| Number of Weeks Looking for Work or on Layoff at any Time in Past Year, Among Those Who Report Looking for Leaving Job Owing to Illness | 2.56 (7.70) | 2.13 (6.76) | 4.71 (11.05) | <.001 | 2.09 (6.68) | 3.95 (10.00) | <.001 |
| Number of Weeks Not Working and Not Looking For Work, Among Those Who Report Looking for Leaving Job Owing to Illness | 17.09 (15.64) | 15.97 (15.13) | 22.61 (16.90) | <.001 | 15.44 (14.88) | 21.94 (16.79) | <.001 |
| Any Unemployment Insurance Income, Among Those Who Report Looking for Leaving Job Owing to Illness | 4.68 (146651.94) | 5.21 (136093.13) | 2.01 (10558.81) | <.001 | 4.66 (109027.96) | 4.73 (37623.98) | 0.619 |
| Amount of Unemployment Insurance Income, if any, $, Among Those Who Report Looking for Leaving Job Owing to Illness | 3946.79 (3785.27) | 3940.47 (3691.71) | 4028.23 (4840.40) | 0.853 | 4242.49 (3878.26) | 3089.89 (3362.14) | 0.035 |
| Any Disability Income, Among Those Who Report Looking for Leaving Job Owing to Illness | 29.30 (919169.45) | 31.89 (832647.15) | 16.45 (86522.30) | <.001 | 31.60 (739803.19) | 22.55 (179366.26) | <.001 |
| Total Amount of Disability Income, if any, $, Among Those Who Report Looking for Leaving Job Owing to Illness | 14056.00 (14242.92) | 14786.88 (14484.37) | 7039.88 (9075.80) | <.001 | 15590.78 (15095.76) | 7743.49 (7117.20) | <.001 |
| Any Worker's Compensation Income, Among Those Who Report Looking for Leaving Job Owing to Illness | 5.83 (182736.56) | 5.98 (156161.01) | 5.05 (26575.55) | 0.388 | 6.29 (147174.57) | 4.47 (35561.99) | 0.009 |
| Amount of Worker's Compensation Income, if any, $, Among Those Who Report Looking for Leaving Job Owing to Illness | 8572.43 (10767.44) | 9547.94 (11228.59) | 2840.22 (4242.54) | <.001 | 9841.82 (11479.74) | 3319.04 (4025.09) | <.001 |
| All numbers weighted to be nationally-representative  P-values are from t-tests for continuous variables and chi-squared tests for categorical variables | | | | | | | |

| Table S10: Demographics, 2022 ASEC Data | | | | | | | |
| --- | --- | --- | --- | --- | --- | --- | --- |
|  | Overall | Poverty Under Absolute Threshold | | | Poverty Under Relative Threshold | | |
|  |  | Not in Poverty | In Poverty | p-value | Not in Poverty | In Poverty | p-value |
| Weighted N | 320130791 | 295236364 | 24894427 |  | 271720381 | 48410410 |  |
|  | % (weighted N) or mean (SD) | % (weighted N) or mean (SD) | % (weighted N) or mean (SD) |  | % (weighted N) or mean (SD) | % (weighted N) or mean (SD) |  |
|  | 39.72 (23.12) | 39.36 (23.04) | 44.03 (23.62) | <.001 | 38.72 (22.72) | 45.36 (24.47) | <.001 |
| Age, y | 51.07 (163502097.31) | 50.96 (150464749.02) | 52.37 (13037348.29) | 0.003 | 50.59 (137453629.19) | 53.81 (26048468.12) | <.001 |
| Female | 320130791 | 295236364 | 24894427 |  | 271720381 | 48410410 |  |
| Race and Ethnicity | |  |  | <.001 |  |  | <.001 |
| Non-Hispanic White | 59.92 (191813111.52) | 61.24 (180815805.03) | 44.18 (10997306.49) |  | 61.56 (167277447.79) | 50.68 (24535663.73) |  |
| Non-Hispanic Black | 11.97 (38328684.70) | 11.52 (34000577.78) | 17.39 (4328106.92) |  | 10.89 (29596089.80) | 18.04 (8732594.90) |  |
| Non-Hispanic American Indian and Alaskan Native | 0.83 (2642072.80) | 0.78 (2304428.19) | 1.36 (337644.61) |  | 0.75 (2025070.98) | 1.27 (617001.82) |  |
| Non-Hispanic Asian | 6.20 (19842573.30) | 6.08 (17943387.23) | 7.63 (1899186.07) |  | 6.40 (17395923.60) | 5.05 (2446649.70) |  |
| Non-Hispanic Native Hawaiian and Other Pacific Islander | 0.32 (1018554.96) | 0.30 (889480.70) | 0.52 (129074.26) |  | 0.32 (868730.57) | 0.31 (149824.39) |  |
| Non-Hispanic Multi-Racial | 1.94 (6204878.48) | 1.95 (5755702.32) | 1.80 (449176.16) |  | 1.93 (5232497.98) | 2.01 (972380.50) |  |
| Hispanic, any race | 18.83 (60280915.41) | 18.13 (53526983.12) | 27.13 (6753932.29) |  | 18.15 (49324620.24) | 22.63 (10956295.17) |  |
| Education |  |  |  | <.001 |  |  | <.001 |
| Child, education incomplete | 18.14 (58056765.24) | 18.67 (55132170.04) | 11.75 (2924595.20) |  | 19.02 (51682693.72) | 13.17 (6374071.52) |  |
| Less than HS Diploma | 11.10 (35524220.77) | 10.29 (30393076.07) | 20.61 (5131144.70) |  | 9.63 (26176013.79) | 19.31 (9348206.98) |  |
| HS Diploma or GED | 22.66 (72556397.79) | 22.02 (65013245.88) | 30.30 (7543151.91) |  | 20.97 (56988631.97) | 32.16 (15567765.82) |  |
| Greater than HS Diploma | 48.10 (153993407.37) | 49.01 (144697872.38) | 37.34 (9295534.99) |  | 50.37 (136873041.48) | 35.37 (17120365.89) |  |
| Marital Status |  |  |  | <.001 |  |  | <.001 |
| Married - Civilian Spouse Present | 39.70 (127102599.78) | 40.93 (120847627.73) | 25.13 (6254972.05) |  | 42.30 (114948815.97) | 25.11 (12153783.81) |  |
| Married - Armed Forces Spouse Present | 0.23 (750678.96) | 0.25 (736873.59) | 0.06 (13805.37) |  | 0.26 (719469.45) | 0.06 (31209.51) |  |
| Married - Spouse Absent | 1.18 (3783505.64) | 1.10 (3258296.18) | 2.11 (525209.46) |  | 1.05 (2856519.32) | 1.91 (926986.32) |  |
| Widowed | 4.81 (15408177.07) | 4.48 (13222016.26) | 8.78 (2186160.81) |  | 3.80 (10312467.78) | 10.53 (5095709.29) |  |
| Divorced | 7.88 (25210766.06) | 7.54 (22265949.55) | 11.83 (2944816.51) |  | 6.95 (18878926.80) | 13.08 (6331839.26) |  |
| Separated | 1.40 (4473155.40) | 1.30 (3846689.25) | 2.52 (626466.15) |  | 1.18 (3212301.79) | 2.60 (1260853.61) |  |
| Never married | 44.79 (143401908.26) | 44.39 (131058911.81) | 49.58 (12342996.45) |  | 44.45 (120791879.85) | 46.70 (22610028.41) |  |
| Personal Factor Income, $ | 34914.59 (76290.78) | 37343.74 (77749.75) | 6105.94 (47489.76) | <.001 | 40074.05 (80383.32) | 5955.28 (35119.45) | <.001 |
| No Personal Factor Income | 36.19 | 34.29 | 58.85 | <.001 | 32.67 | 56.02 | <.001 |
| SPM Resources | 89777.26 (83286.94) | 96192.93 (83455.66) | 13690.49 (18048.78) | <.001 | 102343.12 (84184.90) | 19246.98 (15226.94) | <.001 |
| Household Size | 3.19 (1.71) | 3.25 (1.71) | 2.51 (1.64) | <.001 | 3.32 (1.71) | 2.43 (1.54) | <.001 |
| Number Under Age 18 in Household | 1.00 (1.33) | 1.03 (1.34) | 0.61 (1.12) | <.001 | 1.06 (1.35) | 0.66 (1.14) | <.001 |
| Percent Under Age 18 in Household | 0.22 (0.26) | 0.23 (0.26) | 0.15 (0.24) | <.001 | 0.23 (0.25) | 0.16 (0.25) | <.001 |
| Number Age 65 or Older in Household | 0.36 (0.67) | 0.36 (0.67) | 0.43 (0.69) | <.001 | 0.34 (0.67) | 0.46 (0.70) | <.001 |
| Percent Age 65 or Older in Household | 0.18 (0.34) | 0.17 (0.34) | 0.24 (0.39) | <.001 | 0.16 (0.33) | 0.28 (0.42) | <.001 |
| Number of Working Age Adults in Household | 1.83 (1.13) | 1.86 (1.12) | 1.47 (1.16) | <.001 | 1.92 (1.12) | 1.31 (1.04) | <.001 |
| Percent of Working Age Adults in Household | 0.60 (0.34) | 0.60 (0.33) | 0.61 (0.38) | 0.007 | 0.61 (0.32) | 0.56 (0.39) | <.001 |
| Number of Paid Laborers in Household | 1.50 (1.02) | 1.58 (1.00) | 0.56 (0.76) | <.001 | 1.66 (0.99) | 0.60 (0.71) | <.001 |
| Percent of Paid Laborers in Household | 0.51 (0.35) | 0.53 (0.34) | 0.24 (0.34) | <.001 | 0.55 (0.33) | 0.27 (0.35) | <.001 |
| Number of Disabled Working Age Adults in Household | 0.09 (0.33) | 0.08 (0.30) | 0.24 (0.52) | <.001 | 0.07 (0.28) | 0.22 (0.50) | <.001 |
| Percent of Disabled Working Age Adults in Household | 0.04 (0.14) | 0.03 (0.13) | 0.11 (0.26) | <.001 | 0.02 (0.10) | 0.11 (0.27) | <.001 |
| Out of Pocket Healthcare Expenditures Per Household Member, $ | 2116.20 (2772.49) | 2143.53 (2575.70) | 1792.07 (4478.44) | <.001 | 2195.31 (2578.40) | 1672.19 (3644.74) | <.001 |
| Role |  |  |  | <.001 |  |  | <.001 |
| Child | 22.02 (70508546.67) | 22.64 (66846481.97) | 14.71 (3662064.70) |  | 23.08 (62702213.87) | 16.13 (7806332.80) |  |
| Older Adult | 17.72 (56742717.00) | 17.17 (50706328.68) | 24.25 (6036388.32) |  | 15.91 (43219061.33) | 27.94 (13523655.67) |  |
| Disabled | 3.56 (11403449.76) | 2.92 (8630127.64) | 11.14 (2773322.12) |  | 2.21 (6017977.98) | 11.12 (5385471.78) |  |
| Student | 2.52 (8057268.88) | 2.12 (6265541.90) | 7.20 (1791726.98) |  | 2.15 (5834831.79) | 4.59 (2222437.09) |  |
| Caregiver | 3.89 (12446171.04) | 3.55 (10480812.25) | 7.89 (1965358.79) |  | 3.46 (9405688.60) | 6.28 (3040482.44) |  |
| Unemployed | 0.70 (2240328.82) | 0.49 (1435602.73) | 3.23 (804726.09) |  | 0.46 (1241265.35) | 2.06 (999063.47) |  |
| Other Not In Labor Force | 3.33 (10652962.48) | 2.84 (8372654.68) | 9.16 (2280307.80) |  | 2.63 (7146630.41) | 7.24 (3506332.07) |  |
| Paid Laborer | 46.26 (148079346.52) | 48.27 (142498814.52) | 22.42 (5580532.00) |  | 50.11 (136152711.63) | 24.64 (11926634.89) |  |
| N and percentages weighted to be nationally-representative  P-values are from t-tests for continuous variables and chi-squared tests for categorical variables | | | | | | | |

| Table S11: Poverty Risk by Role, 2022 ASEC Data | | | |
| --- | --- | --- | --- |
|  | Poverty by Absolute Threshold | Relative Risk | P |
|  | % (weighted N) | (95% CI) |  |
| Overall | 7.78 (24894427) | -- | -- |
| Child | 5.19 (3662065) | 1.38 (1.32 to 1.44) | <.001 |
| Older Adult | 10.64 (6036388) | 2.82 (2.72 to 2.93) | <.001 |
| Disabled | 24.32 (2773322) | 6.45 (6.21 to 6.71) | <.001 |
| Student | 22.24 (1791727) | 5.90 (5.38 to 6.47) | <.001 |
| Caregiver | 15.79 (1965359) | 4.19 (3.98 to 4.42) | <.001 |
| Unemployed | 35.92 (804726) | 9.53 (8.97 to 10.13) | <.001 |
| Other Not In Labor Force | 21.41 (2280308) | 5.68 (5.43 to 5.94) | <.001 |
| Paid Laborer | 3.77 (5580532) | ref | ref |
|  |  |  |  |
|  | Poverty by Relative Threshold | Relative Risk |  |
|  | % (weighted N) | (95% CI) |  |
| Overall | 15.12 (48410410) | -- | -- |
| Child | 11.07 (7806333) | 1.37 (1.33 to 1.42) | <.001 |
| Older Adult | 23.83 (13523656) | 2.96 (2.88 to 3.04) | <.001 |
| Disabled | 47.23 (5385472) | 5.86 (5.69 to 6.04) | <.001 |
| Student | 27.58 (2222437) | 3.42 (3.19 to 3.68) | <.001 |
| Caregiver | 24.43 (3040482) | 3.03 (2.92 to 3.15) | <.001 |
| Unemployed | 44.59 (999063) | 5.54 (5.29 to 5.80) | <.001 |
| Other Not In Labor Force | 32.91 (3506332) | 4.09 (3.95 to 4.23) | <.001 |
| Paid Laborer | 8.05 (11926635) | ref | ref |
| N and percent weighted to be nationally-representative.  Relative risk represents risk of experiencing poverty by a given threshold, compared with the risk observed for paid laborers  p-values are from predictive margins using delta-method standard errors are fitting a logistic regression model | | | |

| Table S12: Demographics by Role, 2022 ASEC Data | | | | | | | |
| --- | --- | --- | --- | --- | --- | --- | --- |
| Child | | | | | | | |
|  | Overall | Poverty Under Absolute Threshold | | | Poverty Under Relative Threshold | | |
|  |  | Not in Poverty | In Poverty | p-value | Not in Poverty | In Poverty | p-value |
| Weighted N | 70508547 | 66846482 | 3662065 |  | 62702214 | 7806333 |  |
|  | % or mean (SD) | % or mean (SD) | % or mean (SD) |  | % or mean (SD) | % or mean (SD) |  |
| Age, y | 8.85 (5.09) | 8.84 (5.08) | 8.90 (5.25) | 0.417 | 8.86 (5.08) | 8.75 (5.19) | <.001 |
| Female | 48.83 | 48.83 | 48.89 | 0.939 | 48.76 | 49.40 | <.001 |
| Race and Ethnicity | |  |  | <.001 |  |  | <.001 |
| Non-Hispanic White | 50.71 | 52.03 | 26.50 |  | 52.94 | 32.75 |  |
| Non-Hispanic Black | 13.29 | 12.83 | 21.76 |  | 12.08 | 22.97 |  |
| Non-Hispanic American Indian and Alaskan Native | 1.04 | 0.98 | 2.22 |  | 0.94 | 1.84 |  |
| Non-Hispanic Asian | 5.52 | 5.53 | 5.22 |  | 5.76 | 3.51 |  |
| Non-Hispanic Native Hawaiian and Other Pacific Islander | 0.34 | 0.33 | 0.63 |  | 0.34 | 0.36 |  |
| Non-Hispanic Multi-Racial | 4.12 | 4.13 | 3.96 |  | 4.01 | 4.97 |  |
| Hispanic, any race | 24.98 | 24.18 | 39.71 |  | 23.91 | 33.58 |  |
| Education | |  |  | -- |  |  | -- |
| Child, education incomplete | -- | -- | -- |  | -- | -- |  |
| Less than HS Diploma | -- | -- | -- |  | -- | -- |  |
| HS Diploma or GED | -- | -- | -- |  | -- | -- |  |
| Greater than HS Diploma | -- | -- | -- |  | -- | -- |  |
| Marital Status | |  |  | <.001 |  |  | <.001 |
| Married - Civilian Spouse Present | 0.03 | 0.03 | 0.03 |  | 0.03 | 0.02 |  |
| Married - Armed Forces Spouse Present | 0.00 | 0.00 | 0.00 |  | 0.00 | 0.00 |  |
| Married - Spouse Absent | 0.15 | 0.14 | 0.15 |  | 0.15 | 0.10 |  |
| Widowed | 0.01 | 0.01 | 0.06 |  | 0.01 | 0.06 |  |
| Divorced | 0.04 | 0.04 | 0.00 |  | 0.04 | 0.02 |  |
| Separated | 0.09 | 0.10 | 0.01 |  | 0.09 | 0.11 |  |
| Never married | 99.68 | 99.68 | 99.74 |  | 99.68 | 99.68 |  |
| Personal Factor Income, $ | 383.04 (8908.27) | 398.06 (9144.73) | 108.85 (1165.94) | <.001 | 418.05 (9437.34) | 101.78 (1144.70) | <.001 |
| No Personal Factor Income | 94.99 | 94.85 | 97.49 | <.001 | 94.96 | 97.67 | <.001 |
| SPM Resources, $ | 100243.48 (82800.95) | 104541.35 (82800.38) | 21791.15 (19130.36) | <.001 | 109153.86 (83446.79) | 28673.24 (15285.26) | <.001 |
| Household Size | 4.54 (1.53) | 4.56 (1.52) | 4.16 (1.69) | <.001 | 4.60 (1.53) | 4.09 (1.50) | <.001 |
| Number Under Age 18 in Household | 2.41 (1.26) | 2.42 (1.26) | 2.25 (1.27) | <.001 | 2.43 (1.27) | 2.29 (1.20) | <.001 |
| Percent Under Age 18 in Household | 0.52 (0.14) | 0.52 (0.14) | 0.54 (0.17) | <.001 | 0.51 (0.14) | 0.55 (0.16) | <.001 |
| Number Age 65 or Older in Household | 0.08 (0.33) | 0.08 (0.33) | 0.10 (0.34) | 0.051 | 0.08 (0.33) | 0.09 (0.33) | 0.078 |
| Percent Age 65 or Older in Household | 0.02 (0.07) | 0.02 (0.07) | 0.02 (0.09) | 0.005 | 0.02 (0.07) | 0.02 (0.09) | 0.002 |
| Number of Working Age Adults in Household | 2.05 (0.79) | 2.06 (0.78) | 1.81 (0.99) | <.001 | 2.09 (0.77) | 1.71 (0.83) | <.001 |
| Percent of Working Age Adults in Household | 0.47 (0.15) | 0.47 (0.15) | 0.44 (0.17) | <.001 | 0.47 (0.15) | 0.43 (0.16) | <.001 |
| Number of Paid Laborers in Household | 1.67 (0.85) | 1.73 (0.83) | 0.69 (0.76) | <.001 | 1.78 (0.81) | 0.83 (0.72) | <.001 |
| Percent of Paid Laborers in Household | 0.39 (0.19) | 0.40 (0.19) | 0.18 (0.20) | <.001 | 0.41 (0.18) | 0.22 (0.20) | <.001 |
| Number of Disabled Working Age Adults in Household | 0.06 (0.26) | 0.05 (0.24) | 0.21 (0.50) | <.001 | 0.04 (0.22) | 0.19 (0.46) | <.001 |
| Percent of Disabled Working Age Adults in Household | 0.01 (0.06) | 0.01 (0.06) | 0.06 (0.13) | <.001 | 0.01 (0.05) | 0.05 (0.12) | <.001 |
| Out of Pocket Healthcare Expenditures Per Household Member, $ | 1314.09 (1801.94) | 1326.03 (1656.40) | 1096.14 (3520.02) | 0.001 | 1364.05 (1662.51) | 912.76 (2635.75) | <.001 |
|  |  |  |  |  |  |  |  |
| Older Adult | | | | | | | |
|  | Overall | Poverty Under Absolute Threshold | | | Poverty Under Relative Threshold | | |
|  |  | Not in Poverty | In Poverty | p-value | Not in Poverty | In Poverty | p-value |
| Weighted N | 56742717 | 50706329 | 6036388 |  | 43219061 | 13523656 |  |
|  | % or mean (SD) | % or mean (SD) | % or mean (SD) |  | % or mean (SD) | % or mean (SD) |  |
| Age, y | 73.47 (6.09) | 73.39 (6.03) | 74.21 (6.51) | <.001 | 73.17 (5.96) | 74.45 (6.38) | <.001 |
| Female | 54.35 | 53.72 | 59.63 | <.001 | 52.73 | 59.53 | <.001 |
| Race and Ethnicity | |  |  | <.001 |  |  | <.001 |
| Non-Hispanic White | 74.52 | 76.34 | 59.27 |  | 77.24 | 65.84 |  |
| Non-Hispanic Black | 9.86 | 9.20 | 15.38 |  | 8.32 | 14.77 |  |
| Non-Hispanic American Indian and Alaskan Native | 0.55 | 0.54 | 0.68 |  | 0.46 | 0.84 |  |
| Non-Hispanic Asian | 5.09 | 4.82 | 7.32 |  | 5.27 | 4.52 |  |
| Non-Hispanic Native Hawaiian and Other Pacific Islander | 0.11 | 0.09 | 0.27 |  | 0.09 | 0.18 |  |
| Non-Hispanic Multi-Racial | 0.83 | 0.82 | 0.89 |  | 0.80 | 0.92 |  |
| Hispanic, any race | 9.04 | 8.19 | 16.18 |  | 7.82 | 12.94 |  |
| Education | |  |  | <.001 |  |  | <.001 |
| Child, education incomplete | 0.00 | 0.00 | 0.00 |  | 0.00 | 0.00 |  |
| Less than HS Diploma | 10.79 | 9.31 | 23.25 |  | 7.75 | 20.52 |  |
| HS Diploma or GED | 31.46 | 31.10 | 34.53 |  | 29.46 | 37.86 |  |
| Greater than HS Diploma | 57.75 | 59.59 | 42.22 |  | 62.79 | 41.62 |  |
| Marital Status | |  |  | <.001 |  |  | <.001 |
| Married - Civilian Spouse Present | 56.02 | 57.91 | 40.15 |  | 61.92 | 37.17 |  |
| Married - Armed Forces Spouse Present | 0.01 | 0.01 | 0.00 |  | 0.01 | 0.00 |  |
| Married - Spouse Absent | 1.33 | 1.21 | 2.28 |  | 1.16 | 1.87 |  |
| Widowed | 21.48 | 20.60 | 28.88 |  | 18.55 | 30.82 |  |
| Divorced | 13.39 | 12.99 | 16.72 |  | 11.74 | 18.65 |  |
| Separated | 1.15 | 1.05 | 1.99 |  | 0.97 | 1.71 |  |
| Never married | 6.63 | 6.24 | 9.98 |  | 5.65 | 9.77 |  |
| Personal Factor Income, $ | 19432.74 (74991.62) | 21041.21 (76168.37) | 5921.37 (62663.98) | <.001 | 24423.97 (81870.35) | 3481.69 (42916.97) | <.001 |
| No Personal Factor Income | 31.81 | 28.74 | 57.64 | <.001 | 24.29 | 55.85 | <.001 |
| SPM Resources, $ | 68526.45 (74980.40) | 75613.54 (75995.82) | 8994.09 (19195.51) | <.001 | 85175.64 (78433.04) | 15318.77 (14577.93) | <.001 |
| Household Size | 2.05 (1.12) | 2.08 (1.14) | 1.79 (0.96) | <.001 | 2.17 (1.17) | 1.66 (0.86) | <.001 |
| Number Under Age 18 in Household | 0.10 (0.48) | 0.11 (0.49) | 0.06 (0.33) | <.001 | 0.12 (0.51) | 0.05 (0.33) | <.001 |
| Percent Under Age 18 in Household | 0.02 (0.09) | 0.02 (0.09) | 0.01 (0.07) | <.001 | 0.02 (0.09) | 0.01 (0.07) | <.001 |
| Number Age 65 or Older in Household | 1.53 (0.53) | 1.54 (0.53) | 1.41 (0.53) | <.001 | 1.57 (0.53) | 1.37 (0.51) | <.001 |
| Percent Age 65 or Older in Household | 0.84 (0.26) | 0.84 (0.26) | 0.87 (0.23) | <.001 | 0.83 (0.27) | 0.90 (0.21) | <.001 |
| Number of Working Age Adults in Household | 0.42 (0.80) | 0.43 (0.82) | 0.32 (0.68) | <.001 | 0.48 (0.86) | 0.23 (0.55) | <.001 |
| Percent of Working Age Adults in Household | 0.13 (0.23) | 0.14 (0.23) | 0.11 (0.21) | <.001 | 0.15 (0.23) | 0.09 (0.19) | <.001 |
| Number of Paid Laborers in Household | 0.60 (0.86) | 0.65 (0.88) | 0.16 (0.44) | <.001 | 0.74 (0.91) | 0.14 (0.39) | <.001 |
| Percent of Paid Laborers in Household | 0.26 (0.36) | 0.28 (0.36) | 0.07 (0.21) | <.001 | 0.31 (0.37) | 0.07 (0.21) | <.001 |
| Number of Disabled Working Age Adults in Household | 0.04 (0.22) | 0.04 (0.21) | 0.07 (0.30) | <.001 | 0.04 (0.21) | 0.06 (0.26) | <.001 |
| Percent of Disabled Working Age Adults in Household | 0.02 (0.08) | 0.01 (0.07) | 0.03 (0.11) | <.001 | 0.01 (0.07) | 0.02 (0.10) | <.001 |
| Out of Pocket Healthcare Expenditures Per Household Member, $ | 3515.65 (3691.72) | 3611.18 (3260.67) | 2713.14 (6172.12) | <.001 | 3814.93 (3258.64) | 2559.21 (4695.81) | <.001 |
|  |  |  |  |  |  |  |  |
| Disabled | | | | | | | |
|  | Overall | Poverty Under Absolute Threshold | | | Poverty Under Relative Threshold | | |
|  |  | Not in Poverty | In Poverty | p-value | Not in Poverty | In Poverty | p-value |
| Weighted N | 11403450 | 8630128 | 2773322 |  | 6017978 | 5385472 |  |
|  | % or mean (SD) | % or mean (SD) | % or mean (SD) |  | % or mean (SD) | % or mean (SD) |  |
| Age, y | 48.64 (12.60) | 48.59 (12.70) | 48.80 (12.28) | 0.493 | 47.62 (12.99) | 49.78 (12.05) | <.001 |
| Female | 51.00 | 52.07 | 47.66 | <.001 | 52.24 | 49.61 | 0.0495 |
| Race and Ethnicity | |  |  | <.001 |  |  | <.001 |
| Non-Hispanic White | 56.79 | 58.90 | 50.22 |  | 58.89 | 54.44 |  |
| Non-Hispanic Black | 21.17 | 21.18 | 21.13 |  | 18.82 | 23.79 |  |
| Non-Hispanic American Indian and Alaskan Native | 1.48 | 1.38 | 1.81 |  | 1.36 | 1.61 |  |
| Non-Hispanic Asian | 2.52 | 2.35 | 3.04 |  | 3.26 | 1.69 |  |
| Non-Hispanic Native Hawaiian and Other Pacific Islander | 0.40 | 0.29 | 0.73 |  | 0.41 | 0.39 |  |
| Non-Hispanic Multi-Racial | 1.85 | 1.95 | 1.53 |  | 2.09 | 1.58 |  |
| Hispanic, any race | 15.80 | 13.96 | 21.54 |  | 15.17 | 16.50 |  |
| Education | |  |  | <.001 |  |  | <.001 |
| Child, education incomplete | 0.00 | 0.00 | 0.00 |  | 0.00 | 0.00 |  |
| Less than HS Diploma | 22.38 | 20.35 | 28.67 |  | 17.50 | 27.83 |  |
| HS Diploma or GED | 44.33 | 45.63 | 40.29 |  | 46.49 | 41.91 |  |
| Greater than HS Diploma | 33.29 | 34.02 | 31.03 |  | 36.01 | 30.26 |  |
| Marital Status | |  |  | <.001 |  |  | <.001 |
| Married - Civilian Spouse Present | 27.46 | 29.58 | 20.86 |  | 34.64 | 19.43 |  |
| Married - Armed Forces Spouse Present | 0.11 | 0.14 | 0.00 |  | 0.21 | 0.00 |  |
| Married - Spouse Absent | 1.93 | 1.64 | 2.82 |  | 1.29 | 2.64 |  |
| Widowed | 4.79 | 4.37 | 6.12 |  | 3.53 | 6.21 |  |
| Divorced | 20.47 | 19.97 | 22.05 |  | 17.46 | 23.83 |  |
| Separated | 3.90 | 3.80 | 4.20 |  | 3.07 | 4.82 |  |
| Never married | 41.34 | 40.51 | 43.95 |  | 39.80 | 43.07 |  |
| Personal Factor Income, $ | 328.07 (4274.82) | 379.23 (4586.90) | 168.86 (3105.55) | 0.004 | 528.49 (5480.76) | 104.10 (2244.83) | <.001 |
| No Personal Factor Income | 79.95 | 78.57 | 84.26 | <.001 | 76.21 | 84.13 | <.001 |
| SPM Resources, $ | 44812.61 (46977.45) | 55125.36 (49383.82) | 12721.01 (11168.93) | <.001 | 69518.08 (52816.80) | 17205.56 (10572.68) | <.001 |
| Household Size | 2.52 (1.52) | 2.64 (1.54) | 2.13 (1.35) | <.001 | 2.98 (1.57) | 2.00 (1.27) | <.001 |
| Number Under Age 18 in Household | 0.36 (0.84) | 0.39 (0.88) | 0.28 (0.70) | <.001 | 0.44 (0.92) | 0.27 (0.73) | <.001 |
| Percent Under Age 18 in Household | 0.08 (0.17) | 0.09 (0.18) | 0.07 (0.17) | 0.001 | 0.09 (0.18) | 0.07 (0.17) | <.001 |
| Number Age 65 or Older in Household | 0.21 (0.48) | 0.23 (0.50) | 0.16 (0.42) | <.001 | 0.27 (0.54) | 0.15 (0.40) | <.001 |
| Percent Age 65 or Older in Household | 0.08 (0.17) | 0.08 (0.18) | 0.06 (0.15) | <.001 | 0.09 (0.18) | 0.06 (0.15) | <.001 |
| Number of Working Age Adults in Household | 1.94 (1.03) | 2.03 (1.04) | 1.68 (0.93) | <.001 | 2.26 (1.08) | 1.58 (0.83) | <.001 |
| Percent of Working Age Adults in Household | 0.84 (0.23) | 0.83 (0.23) | 0.87 (0.21) | <.001 | 0.81 (0.23) | 0.87 (0.22) | <.001 |
| Number of Paid Laborers in Household | 0.61 (0.82) | 0.74 (0.86) | 0.19 (0.50) | <.001 | 0.97 (0.89) | 0.20 (0.46) | <.001 |
| Percent of Paid Laborers in Household | 0.19 (0.24) | 0.23 (0.24) | 0.06 (0.16) | <.001 | 0.30 (0.24) | 0.07 (0.16) | <.001 |
| Number of Disabled Working Age Adults in Household | 1.19 (0.46) | 1.18 (0.44) | 1.24 (0.52) | 0.001 | 1.18 (0.44) | 1.21 (0.48) | 0.026 |
| Percent of Disabled Working Age Adults in Household | 0.61 (0.30) | 0.58 (0.30) | 0.73 (0.30) | <.001 | 0.49 (0.26) | 0.75 (0.29) | <.001 |
| Out of Pocket Healthcare Expenditures Per Household Member, $ | 1350.14 (2279.76) | 1389.91 (1987.13) | 1226.40 (3011.48) | <.001 | 1678.20 (2158.98) | 983.56 (2354.53) | <.001 |
|  |  |  |  |  |  |  |  |
| Student | | | | | | | |
|  | Overall | Poverty Under Absolute Threshold | | | Poverty Under Relative Threshold | | |
|  |  | Not in Poverty | In Poverty | p-value | Not in Poverty | In Poverty | p-value |
| Weighted N | 8057269 | 6265542 | 1791727 |  | 5834832 | 2222437 |  |
|  | % or mean (SD) | % or mean (SD) | % or mean (SD) |  | % or mean (SD) | % or mean (SD) |  |
| Age, y | 23.17 (7.49) | 22.73 (7.30) | 24.72 (7.92) | <.001 | 22.64 (7.22) | 24.57 (7.99) | <.001 |
| Female | 50.29 | 51.43 | 46.29 | <.001 | 51.72 | 46.53 | <.001 |
| Race and Ethnicity | |  |  | <.001 |  |  | <.001 |
| Non-Hispanic White | 45.18 | 47.61 | 36.70 |  | 48.88 | 35.47 |  |
| Non-Hispanic Black | 14.12 | 13.53 | 16.15 |  | 12.39 | 18.65 |  |
| Non-Hispanic American Indian and Alaskan Native | 1.50 | 1.33 | 2.10 |  | 1.23 | 2.20 |  |
| Non-Hispanic Asian | 12.33 | 10.62 | 18.33 |  | 10.85 | 16.23 |  |
| Non-Hispanic Native Hawaiian and Other Pacific Islander | 0.38 | 0.37 | 0.43 |  | 0.38 | 0.37 |  |
| Non-Hispanic Multi-Racial | 2.40 | 2.35 | 2.56 |  | 2.26 | 2.76 |  |
| Hispanic, any race | 24.09 | 24.20 | 23.73 |  | 24.01 | 24.31 |  |
| Education | |  |  | <.001 |  |  | <.001 |
| Child, education incomplete | 0.00 | 0.00 | 0.00 |  | 0.00 | 0.00 |  |
| Less than HS Diploma | 23.27 | 25.53 | 15.36 |  | 25.07 | 18.52 |  |
| HS Diploma or GED | 17.49 | 16.55 | 20.80 |  | 15.94 | 21.57 |  |
| Greater than HS Diploma | 59.24 | 57.93 | 63.83 |  | 58.99 | 59.91 |  |
| Marital Status | |  |  | <.001 |  |  | <.001 |
| Married - Civilian Spouse Present | 6.71 | 6.92 | 6.01 |  | 6.69 | 6.78 |  |
| Married - Armed Forces Spouse Present | 0.51 | 0.65 | 0.00 |  | 0.62 | 0.22 |  |
| Married - Spouse Absent | 0.64 | 0.62 | 0.72 |  | 0.48 | 1.07 |  |
| Widowed | 0.34 | 0.18 | 0.91 |  | 0.13 | 0.90 |  |
| Divorced | 1.01 | 0.74 | 1.94 |  | 0.70 | 1.81 |  |
| Separated | 0.43 | 0.43 | 0.43 |  | 0.30 | 0.79 |  |
| Never married | 90.35 | 90.46 | 89.99 |  | 91.09 | 88.43 |  |
| Personal Factor Income, $ | 104.09 (2621.54) | 123.81 (2949.04) | 35.13 (699.36) | 0.003 | 127.99 (3053.14) | 41.35 (662.90) | 0.003 |
| No Personal Factor Income | 81.47 | 82.38 | 78.27 | <.001 | 82.07 | 79.89 | 0.046 |
| SPM Resources, $ | 83023.58 (95626.33) | 103216.18 (99310.79) | 12411.52 (14889.60) | <.001 | 108607.01 (100828.33) | 15856.33 (15248.82) | <.001 |
| Household Size | 3.65 (1.80) | 4.02 (1.65) | 2.38 (1.72) | <.001 | 4.07 (1.65) | 2.55 (1.71) | <.001 |
| Number Under Age 18 in Household | 0.67 (1.05) | 0.77 (1.11) | 0.31 (0.72) | <.001 | 0.76 (1.11) | 0.43 (0.85) | <.001 |
| Percent Under Age 18 in Household | 0.13 (0.18) | 0.15 (0.19) | 0.07 (0.15) | <.001 | 0.15 (0.19) | 0.10 (0.18) | <.001 |
| Number Age 65 or Older in Household | 0.14 (0.44) | 0.16 (0.46) | 0.08 (0.33) | <.001 | 0.16 (0.47) | 0.09 (0.34) | <.001 |
| Percent Age 65 or Older in Household | 0.03 (0.11) | 0.04 (0.11) | 0.02 (0.10) | <.001 | 0.04 (0.11) | 0.03 (0.10) | 0.002 |
| Number of Working Age Adults in Household | 2.84 (1.26) | 3.09 (1.14) | 2.00 (1.28) | <.001 | 3.15 (1.13) | 2.03 (1.19) | <.001 |
| Percent of Working Age Adults in Household | 0.83 (0.20) | 0.81 (0.21) | 0.91 (0.18) | <.001 | 0.82 (0.20) | 0.88 (0.20) | <.001 |
| Number of Paid Laborers in Household | 1.34 (1.02) | 1.62 (0.94) | 0.36 (0.65) | <.001 | 1.68 (0.93) | 0.45 (0.66) | <.001 |
| Percent of Paid Laborers in Household | 0.34 (0.23) | 0.41 (0.20) | 0.10 (0.18) | <.001 | 0.42 (0.20) | 0.13 (0.19) | <.001 |
| Number of Disabled Working Age Adults in Household | 0.07 (0.28) | 0.06 (0.26) | 0.11 (0.35) | <.001 | 0.06 (0.26) | 0.11 (0.34) | <.001 |
| Percent of Disabled Working Age Adults in Household | 0.02 (0.08) | 0.02 (0.07) | 0.03 (0.10) | <.001 | 0.02 (0.07) | 0.03 (0.10) | <.001 |
| Out of Pocket Healthcare Expenditures Per Household Member, $ | 1434.97 (1943.62) | 1541.17 (1828.56) | 1063.62 (2263.64) | <.001 | 1596.60 (1848.74) | 1010.63 (2116.06) | <.001 |
|  |  |  |  |  |  |  |  |
| Caregiver | | | | | | | |
|  | Overall | Poverty Under Absolute Threshold | | | Poverty Under Relative Threshold | | |
|  |  | Not in Poverty | In Poverty | p-value | Not in Poverty | In Poverty | p-value |
| Weighted N | 12446171 | 10480812 | 1965359 |  | 9405689 | 3040482 |  |
|  | % or mean (SD) | % or mean (SD) | % or mean (SD) |  | % or mean (SD) | % or mean (SD) |  |
| Age, y | 40.50 (11.54) | 40.56 (11.34) | 40.18 (12.58) | 0.187 | 40.68 (11.22) | 39.93 (12.48) | 0.001 |
| Female | 89.84 | 91.43 | 81.35 | <.001 | 91.87 | 83.54 | <.001 |
| Race and Ethnicity | |  |  | <.001 |  |  | <.001 |
| Non-Hispanic White | 50.51 | 53.21 | 36.13 |  | 53.63 | 40.89 |  |
| Non-Hispanic Black | 8.11 | 6.88 | 14.71 |  | 5.74 | 15.44 |  |
| Non-Hispanic American Indian and Alaskan Native | 0.97 | 0.96 | 1.03 |  | 0.95 | 1.05 |  |
| Non-Hispanic Asian | 8.94 | 9.14 | 7.91 |  | 9.78 | 6.36 |  |
| Non-Hispanic Native Hawaiian and Other Pacific Islander | 0.52 | 0.45 | 0.90 |  | 0.48 | 0.65 |  |
| Non-Hispanic Multi-Racial | 1.18 | 1.20 | 1.05 |  | 1.14 | 1.30 |  |
| Hispanic, any race | 29.75 | 28.16 | 38.27 |  | 28.28 | 34.31 |  |
| Education | |  |  | <.001 |  |  | <.001 |
| Child, education incomplete | 0.00 | 0.00 | 0.00 |  | 0.00 | 0.00 |  |
| Less than HS Diploma | 16.09 | 14.45 | 24.84 |  | 13.48 | 24.18 |  |
| HS Diploma or GED | 32.87 | 31.64 | 39.43 |  | 30.23 | 41.04 |  |
| Greater than HS Diploma | 51.04 | 53.91 | 35.72 |  | 56.29 | 34.78 |  |
| Marital Status | |  |  | <.001 |  |  | <.001 |
| Married - Civilian Spouse Present | 69.51 | 74.95 | 40.50 |  | 77.31 | 45.38 |  |
| Married - Armed Forces Spouse Present | 1.66 | 1.87 | 0.50 |  | 2.02 | 0.53 |  |
| Married - Spouse Absent | 1.41 | 1.08 | 3.21 |  | 1.07 | 2.48 |  |
| Widowed | 0.91 | 0.77 | 1.64 |  | 0.69 | 1.57 |  |
| Divorced | 4.23 | 3.18 | 9.84 |  | 2.75 | 8.83 |  |
| Separated | 1.55 | 1.08 | 4.09 |  | 0.88 | 3.64 |  |
| Never married | 20.73 | 17.07 | 40.22 |  | 15.28 | 37.56 |  |
| Personal Factor Income, $ | 1349.75 (18900.81) | 1527.25 (20349.35) | 403.20 (7286.34) | <.001 | 1671.26 (21362.82) | 355.19 (7026.55) | <.001 |
| No Personal Factor Income | 67.34 | 63.73 | 86.57 | <.001 | 61.56 | 85.21 | <.001 |
| SPM Resources, $ | 81108.88 (83499.48) | 93070.57 (85634.06) | 17319.93 (14674.75) | <.001 | 100256.59 (87559.19) | 21875.71 (13478.95) | <.001 |
| Household Size | 3.94 (1.68) | 4.07 (1.63) | 3.25 (1.82) | <.001 | 4.16 (1.65) | 3.26 (1.59) | <.001 |
| Number Under Age 18 in Household | 1.48 (1.42) | 1.57 (1.42) | 1.02 (1.31) | <.001 | 1.60 (1.44) | 1.12 (1.29) | <.001 |
| Percent Under Age 18 in Household | 0.31 (0.25) | 0.33 (0.24) | 0.23 (0.26) | <.001 | 0.33 (0.24) | 0.26 (0.26) | <.001 |
| Number Age 65 or Older in Household | 0.11 (0.37) | 0.11 (0.37) | 0.13 (0.38) | 0.015 | 0.11 (0.37) | 0.12 (0.36) | 0.25 |
| Percent Age 65 or Older in Household | 0.03 (0.11) | 0.03 (0.11) | 0.04 (0.13) | 0.001 | 0.03 (0.11) | 0.04 (0.13) | 0.008 |
| Number of Working Age Adults in Household | 2.34 (0.96) | 2.39 (0.92) | 2.10 (1.08) | <.001 | 2.45 (0.95) | 2.02 (0.89) | <.001 |
| Percent of Working Age Adults in Household | 0.65 (0.24) | 0.64 (0.24) | 0.73 (0.26) | <.001 | 0.64 (0.23) | 0.70 (0.26) | <.001 |
| Number of Paid Laborers in Household | 1.09 (0.78) | 1.21 (0.74) | 0.49 (0.69) | <.001 | 1.27 (0.75) | 0.54 (0.61) | <.001 |
| Percent of Paid Laborers in Household | 0.29 (0.19) | 0.32 (0.17) | 0.14 (0.19) | <.001 | 0.33 (0.17) | 0.16 (0.19) | <.001 |
| Number of Disabled Working Age Adults in Household | 0.07 (0.27) | 0.05 (0.24) | 0.15 (0.40) | <.001 | 0.04 (0.22) | 0.14 (0.38) | <.001 |
| Percent of Disabled Working Age Adults in Household | 0.02 (0.08) | 0.01 (0.06) | 0.04 (0.12) | <.001 | 0.01 (0.05) | 0.04 (0.12) | <.001 |
| Out of Pocket Healthcare Expenditures Per Household Member, $ | 1378.99 (2077.29) | 1468.55 (2063.16) | 901.35 (2088.21) | <.001 | 1515.13 (2070.79) | 957.84 (2041.20) | <.001 |
|  |  |  |  |  |  |  |  |
| Unemployed | | | | | | | |
|  | Overall | Poverty Under Absolute Threshold | | | Poverty Under Relative Threshold | | |
|  |  | Not in Poverty | In Poverty | p-value | Not in Poverty | In Poverty | p-value |
| Weighted N | 2240329 | 1435603 | 804726 |  | 1241265 | 999063 |  |
|  | % or mean (SD) | % or mean (SD) | % or mean (SD) |  | % or mean (SD) | % or mean (SD) |  |
| Age, y | 38.03 (13.62) | 36.70 (13.93) | 40.41 (12.74) | <.001 | 36.51 (14.00) | 39.92 (12.91) | <.001 |
| Female | 37.92 | 37.99 | 37.80 | 0.934 | 35.89 | 40.43 | 0.017 |
| Race and Ethnicity | |  |  | <.001 |  |  | <.001 |
| Non-Hispanic White | 41.85 | 46.71 | 33.19 |  | 44.97 | 37.98 |  |
| Non-Hispanic Black | 20.00 | 17.21 | 24.98 |  | 15.82 | 25.19 |  |
| Non-Hispanic American Indian and Alaskan Native | 0.91 | 1.20 | 0.40 |  | 0.81 | 1.03 |  |
| Non-Hispanic Asian | 8.06 | 8.50 | 7.26 |  | 9.40 | 6.39 |  |
| Non-Hispanic Native Hawaiian and Other Pacific Islander | 0.37 | 0.23 | 0.62 |  | 0.27 | 0.50 |  |
| Non-Hispanic Multi-Racial | 2.02 | 2.38 | 1.40 |  | 2.49 | 1.45 |  |
| Hispanic, any race | 26.78 | 23.77 | 32.16 |  | 26.25 | 27.45 |  |
| Education | |  |  | <.001 |  |  | <.001 |
| Child, education incomplete | 0.00 | 0.00 | 0.00 |  | 0.00 | 0.00 |  |
| Less than HS Diploma | 14.33 | 12.68 | 17.29 |  | 12.86 | 16.17 |  |
| HS Diploma or GED | 44.72 | 42.41 | 48.85 |  | 42.31 | 47.71 |  |
| Greater than HS Diploma | 40.94 | 44.91 | 33.86 |  | 44.83 | 36.12 |  |
| Marital Status | |  |  | <.001 |  |  | <.001 |
| Married - Civilian Spouse Present | 26.99 | 32.29 | 17.53 |  | 33.68 | 18.67 |  |
| Married - Armed Forces Spouse Present | 0.11 | 0.18 | 0.00 |  | 0.20 | 0.00 |  |
| Married - Spouse Absent | 2.02 | 0.93 | 3.96 |  | 0.85 | 3.48 |  |
| Widowed | 1.21 | 1.30 | 1.05 |  | 1.22 | 1.20 |  |
| Divorced | 10.07 | 7.96 | 13.84 |  | 6.07 | 15.05 |  |
| Separated | 2.67 | 1.78 | 4.25 |  | 1.67 | 3.90 |  |
| Never married | 56.93 | 55.56 | 59.36 |  | 56.31 | 57.70 |  |
| Personal Factor Income, $ | 1138.46 (18526.08) | 1028.78 (10598.12) | 1334.11 (27506.14) | 0.619 | 1184.94 (11390.69) | 1080.70 (24684.70) | <.001 |
| No Personal Factor Income | 81.23 | 77.02 | 88.75 | <.001 | 76.07 | 87.64 | <.001 |
| SPM Resources, $ | 52454.10 (62491.49) | 74772.79 (67792.47) | 12638.34 (14199.70) | <.001 | 82403.70 (69869.31) | 15243.84 (13984.19) | <.001 |
| Household Size | 3.08 (1.65) | 3.49 (1.66) | 2.36 (1.36) | <.001 | 3.63 (1.68) | 2.41 (1.35) | <.001 |
| Number Under Age 18 in Household | 0.59 (1.01) | 0.72 (1.11) | 0.36 (0.75) | <.001 | 0.71 (1.11) | 0.44 (0.85) | <.001 |
| Percent Under Age 18 in Household | 0.14 (0.21) | 0.16 (0.22) | 0.10 (0.19) | <.001 | 0.15 (0.21) | 0.12 (0.21) | <.001 |
| Number Age 65 or Older in Household | 0.17 (0.44) | 0.21 (0.49) | 0.11 (0.32) | 0.001 | 0.23 (0.51) | 0.10 (0.31) | <.001 |
| Percent Age 65 or Older in Household | 0.05 (0.13) | 0.06 (0.14) | 0.04 (0.12) | 0.076 | 0.06 (0.15) | 0.03 (0.12) | 0.001 |
| Number of Working Age Adults in Household | 2.32 (1.15) | 2.56 (1.15) | 1.89 (1.03) | <.001 | 2.68 (1.16) | 1.87 (0.98) | <.001 |
| Percent of Working Age Adults in Household | 0.81 (0.23) | 0.79 (0.23) | 0.86 (0.21) | <.001 | 0.79 (0.23) | 0.85 (0.23) | <.001 |
| Number of Paid Laborers in Household | 0.90 (0.94) | 1.23 (0.95) | 0.31 (0.58) | <.001 | 1.33 (0.95) | 0.36 (0.59) | <.001 |
| Percent of Paid Laborers in Household | 0.26 (0.24) | 0.35 (0.22) | 0.10 (0.18) | <.001 | 0.37 (0.22) | 0.12 (0.19) | <.001 |
| Number of Disabled Working Age Adults in Household | 0.11 (0.36) | 0.09 (0.34) | 0.14 (0.41) | 0.028 | 0.08 (0.32) | 0.14 (0.41) | 0.001 |
| Percent of Disabled Working Age Adults in Household | 0.03 (0.11) | 0.03 (0.09) | 0.05 (0.13) | 0.003 | 0.02 (0.08) | 0.05 (0.14) | <.001 |
| Out of Pocket Healthcare Expenditures Per Household Member, $ | 1235.05 (2090.85) | 1480.25 (2213.08) | 797.63 (1773.42) | <.001 | 1562.07 (2195.34) | 828.75 (1877.87) | <.001 |
|  |  |  |  |  |  |  |  |
| Other Not In Labor Force | | | | | | | |
|  | Overall | Poverty Under Absolute Threshold | | | Poverty Under Relative Threshold | | |
|  |  | Not in Poverty | In Poverty | p-value | Not in Poverty | In Poverty | p-value |
| Weighted N | 10652962 | 8372655 | 2280308 |  | 7146630 | 3506332 |  |
|  | % or mean (SD) | % or mean (SD) | % or mean (SD) |  | % or mean (SD) | % or mean (SD) |  |
| Age, y | 51.22 (13.99) | 51.98 (13.75) | 48.45 (14.48) | <.001 | 51.92 (13.78) | 49.79 (14.29) | <.001 |
| Female | 53.88 | 55.60 | 47.57 | <.001 | 55.65 | 50.27 | <.001 |
| Race and Ethnicity | |  |  | <.001 |  |  | <.001 |
| Non-Hispanic White | 64.39 | 67.47 | 53.07 |  | 67.26 | 58.53 |  |
| Non-Hispanic Black | 12.31 | 10.91 | 17.43 |  | 10.19 | 16.61 |  |
| Non-Hispanic American Indian and Alaskan Native | 1.25 | 1.10 | 1.82 |  | 1.03 | 1.71 |  |
| Non-Hispanic Asian | 6.63 | 6.09 | 8.60 |  | 6.74 | 6.41 |  |
| Non-Hispanic Native Hawaiian and Other Pacific Islander | 0.17 | 0.20 | 0.06 |  | 0.23 | 0.04 |  |
| Non-Hispanic Multi-Racial | 1.12 | 1.06 | 1.35 |  | 1.07 | 1.22 |  |
| Hispanic, any race | 14.13 | 13.17 | 17.67 |  | 13.47 | 15.48 |  |
| Education | |  |  | <.001 |  |  | <.001 |
| Child, education incomplete | 0.00 | 0.00 | 0.00 |  | 0.00 | 0.00 |  |
| Less than HS Diploma | 10.27 | 8.70 | 16.00 |  | 8.01 | 14.86 |  |
| HS Diploma or GED | 34.82 | 34.02 | 37.77 |  | 32.31 | 39.95 |  |
| Greater than HS Diploma | 54.91 | 57.28 | 46.23 |  | 59.68 | 45.19 |  |
| Marital Status | |  |  | <.001 |  |  | <.001 |
| Married - Civilian Spouse Present | 55.54 | 60.40 | 37.71 |  | 63.47 | 39.40 |  |
| Married - Armed Forces Spouse Present | 0.11 | 0.14 | 0.03 |  | 0.16 | 0.02 |  |
| Married - Spouse Absent | 1.94 | 1.83 | 2.35 |  | 1.72 | 2.38 |  |
| Widowed | 5.38 | 5.43 | 5.18 |  | 4.60 | 6.97 |  |
| Divorced | 11.46 | 10.27 | 15.83 |  | 9.40 | 15.67 |  |
| Separated | 2.01 | 1.79 | 2.81 |  | 1.69 | 2.66 |  |
| Never married | 23.55 | 20.13 | 36.10 |  | 18.96 | 32.90 |  |
| Personal Factor Income, $ | 8533.67 (53254.46) | 9392.60 (54168.15) | 5379.90 (49655.67) | <.001 | 10804.41 (58488.52) | 3905.43 (40168.44) | <.001 |
| No Personal Factor Income | 45.32 | 41.19 | 60.54 | <.001 | 38.29 | 59.67 | <.001 |
| SPM Resources, $ | 66401.53 (78631.92) | 82042.62 (81645.15) | 8971.79 (14627.66) | <.001 | 92138.33 (84318.60) | 13944.61 (13899.70) | <.001 |
| Household Size | 2.57 (1.50) | 2.66 (1.50) | 2.22 (1.44) | <.001 | 2.78 (1.56) | 2.14 (1.27) | <.001 |
| Number Under Age 18 in Household | 0.32 (0.82) | 0.33 (0.84) | 0.27 (0.74) | <.001 | 0.35 (0.87) | 0.25 (0.69) | <.001 |
| Percent Under Age 18 in Household | 0.07 (0.16) | 0.07 (0.16) | 0.07 (0.16) | 0.235 | 0.07 (0.16) | 0.06 (0.16) | 0.001 |
| Number Age 65 or Older in Household | 0.26 (0.50) | 0.26 (0.50) | 0.23 (0.49) | 0.005 | 0.27 (0.50) | 0.23 (0.49) | <.001 |
| Percent Age 65 or Older in Household | 0.10 (0.19) | 0.10 (0.20) | 0.09 (0.18) | <.001 | 0.11 (0.19) | 0.09 (0.19) | 0.001 |
| Number of Working Age Adults in Household | 1.99 (1.04) | 2.07 (1.05) | 1.72 (0.98) | <.001 | 2.15 (1.09) | 1.66 (0.85) | <.001 |
| Percent of Working Age Adults in Household | 0.83 (0.23) | 0.83 (0.23) | 0.85 (0.23) | <.001 | 0.82 (0.23) | 0.85 (0.23) | <.001 |
| Number of Paid Laborers in Household | 0.65 (0.85) | 0.78 (0.88) | 0.18 (0.46) | <.001 | 0.86 (0.91) | 0.22 (0.48) | <.001 |
| Percent of Paid Laborers in Household | 0.21 (0.24) | 0.25 (0.25) | 0.06 (0.15) | <.001 | 0.28 (0.25) | 0.08 (0.17) | <.001 |
| Number of Disabled Working Age Adults in Household | 0.05 (0.25) | 0.05 (0.24) | 0.06 (0.29) | 0.016 | 0.05 (0.24) | 0.06 (0.28) | 0.002 |
| Percent of Disabled Working Age Adults in Household | 0.02 (0.08) | 0.02 (0.08) | 0.02 (0.10) | 0.001 | 0.01 (0.07) | 0.02 (0.10) | <.001 |
| Out of Pocket Healthcare Expenditures Per Household Member, $ | 2433.19 (3382.76) | 2597.53 (3276.10) | 1829.77 (3688.00) | <.001 | 2741.23 (3347.09) | 1805.34 (3369.32) | <.001 |
|  |  |  |  |  |  |  |  |
| Paid Laborer | | | | | | | |
|  | Overall | Poverty Under Absolute Threshold | | | Poverty Under Relative Threshold | | |
|  |  | Not in Poverty | In Poverty | p-value | Not in Poverty | In Poverty | p-value |
| Weighted N | 148079347 | 142498815 | 5580532 |  | 136152712 | 11926635 |  |
|  | % or mean (SD) | % or mean (SD) | % or mean (SD) |  | % or mean (SD) | % or mean (SD) |  |
| Age, y | 40.84 (12.77) | 40.94 (12.72) | 38.36 (13.90) | <.001 | 41.02 (12.66) | 38.73 (13.77) | <.001 |
| Female | 47.67 | 47.78 | 44.95 | <.001 | 47.64 | 48.04 | 0.023 |
| Race and Ethnicity | |  |  | <.001 |  |  | <.001 |
| Non-Hispanic White | 60.49 | 61.31 | 39.63 |  | 61.62 | 47.63 |  |
| Non-Hispanic Black | 11.51 | 11.37 | 15.05 |  | 11.09 | 16.29 |  |
| Non-Hispanic American Indian and Alaskan Native | 0.69 | 0.68 | 1.12 |  | 0.67 | 1.01 |  |
| Non-Hispanic Asian | 6.61 | 6.56 | 7.94 |  | 6.73 | 5.27 |  |
| Non-Hispanic Native Hawaiian and Other Pacific Islander | 0.37 | 0.36 | 0.68 |  | 0.37 | 0.35 |  |
| Non-Hispanic Multi-Racial | 1.43 | 1.42 | 1.78 |  | 1.40 | 1.82 |  |
| Hispanic, any race | 18.90 | 18.31 | 33.81 |  | 18.13 | 27.64 |  |
| Education | |  |  | <.001 |  |  | <.001 |
| Child, education incomplete | 0.00 | 0.00 | 0.00 |  | 0.00 | 0.00 |  |
| Less than HS Diploma | 6.48 | 6.05 | 17.33 |  | 5.67 | 15.63 |  |
| HS Diploma or GED | 26.45 | 26.14 | 34.25 |  | 25.42 | 38.16 |  |
| Greater than HS Diploma | 67.08 | 67.81 | 48.42 |  | 68.90 | 46.21 |  |
| Marital Status | |  |  | <.001 |  |  | <.001 |
| Married - Civilian Spouse Present | 51.63 | 52.71 | 24.13 |  | 53.96 | 25.00 |  |
| Married - Armed Forces Spouse Present | 0.32 | 0.33 | 0.06 |  | 0.34 | 0.08 |  |
| Married - Spouse Absent | 1.50 | 1.46 | 2.55 |  | 1.41 | 2.57 |  |
| Widowed | 1.30 | 1.28 | 1.71 |  | 1.22 | 2.21 |  |
| Divorced | 8.91 | 8.82 | 11.18 |  | 8.58 | 12.71 |  |
| Separated | 1.90 | 1.83 | 3.64 |  | 1.70 | 4.19 |  |
| Never married | 34.44 | 33.57 | 56.73 |  | 32.79 | 53.24 |  |
| Personal Factor Income, $ | 67077.03 (90194.62) | 68993.74 (90451.39) | 18133.85 (66807.50) | <.001 | 71308.24 (91853.38) | 18774.12 (46375.32) | <.001 |
| No Personal Factor Income | -- | -- | -- | -- | -- | -- | -- |
| SPM Resources, $ | 99741.96 (85272.21) | 103054.79 (85141.11) | 15148.79 (20142.62) | <.001 | 106700.08 (85358.00) | 20309.07 (15556.32) | <.001 |
| Household Size | 2.99 (1.55) | 3.02 (1.55) | 2.34 (1.51) | <.001 | 3.06 (1.55) | 2.27 (1.41) | <.001 |
| Number Under Age 18 in Household | 0.75 (1.10) | 0.77 (1.11) | 0.43 (0.87) | <.001 | 0.77 (1.11) | 0.52 (0.95) | <.001 |
| Percent Under Age 18 in Household | 0.18 (0.23) | 0.18 (0.23) | 0.11 (0.20) | <.001 | 0.18 (0.23) | 0.13 (0.22) | <.001 |
| Number Age 65 or Older in Household | 0.11 (0.36) | 0.11 (0.36) | 0.08 (0.33) | <.001 | 0.11 (0.37) | 0.07 (0.30) | <.001 |
| Percent Age 65 or Older in Household | 0.03 (0.12) | 0.03 (0.12) | 0.03 (0.11) | <.001 | 0.03 (0.12) | 0.03 (0.11) | <.001 |
| Number of Working Age Adults in Household | 2.13 (0.95) | 2.15 (0.95) | 1.82 (1.03) | <.001 | 2.17 (0.95) | 1.68 (0.83) | <.001 |
| Percent of Working Age Adults in Household | 0.79 (0.24) | 0.79 (0.24) | 0.86 (0.22) | <.001 | 0.79 (0.24) | 0.84 (0.24) | <.001 |
| Number of Paid Laborers in Household | 1.94 (0.87) | 1.96 (0.87) | 1.37 (0.65) | <.001 | 2.00 (0.87) | 1.31 (0.55) | <.001 |
| Percent of Paid Laborers in Household | 0.74 (0.27) | 0.74 (0.27) | 0.73 (0.30) | 0.009 | 0.74 (0.26) | 0.72 (0.30) | <.001 |
| Number of Disabled Working Age Adults in Household | 0.04 (0.22) | 0.04 (0.21) | 0.09 (0.32) | <.001 | 0.04 (0.21) | 0.08 (0.30) | <.001 |
| Percent of Disabled Working Age Adults in Household | 0.01 (0.07) | 0.01 (0.07) | 0.03 (0.10) | <.001 | 0.01 (0.07) | 0.03 (0.11) | <.001 |
| Out of Pocket Healthcare Expenditures Per Household Member, $ | 2110.42 (2607.70) | 2106.56 (2472.74) | 2209.14 (4930.34) | 0.146 | 2136.62 (2472.11) | 1811.33 (3816.89) | <.001 |
| All numbers weighted to be nationally-representative  P-values are from t-tests for continuous variables and chi-squared tests for categorical variables | | | | | | | |
